# Ontogenetic and inter-elemental osteohistological variability in the leopard tortoise *Stigmochelys pardalis*

**DOI:** 10.7717/peerj.8030

**Published:** 2019-12-17

**Authors:** Alexander Edward Botha, Jennifer Botha

**Affiliations:** 1 Department of Zoology and Entomology, University of the Free State, Bloemfontein, Free State, South Africa; 2 Department of Karoo Palaeontology, National Museum, Bloemfontein, Free State, South Africa

**Keywords:** Testudines, Osteohistology, Microanatomy, Ontogenetic variability, Inter-elemental variability, Ecological signal, Lifestyle

## Abstract

Testudines are a group of reptiles characterized by the presence of a shell covered by keratinous shields. *Stigmochelys pardalis* is the most widely distributed terrestrial testudine in southern Africa. Although relatively common with some life history traits being well known, the growth of this species has yet to be studied in any detail. The bone microanatomy of this clade differs from that found in other amniotes, where terrestrial species tend to display characteristics normally seen in aquatic species and vice versa. A detailed histological analysis of the limb bones of *S. pardalis* reveals extensive variation through ontogeny. Cortical bone becomes increasingly thicker through ontogeny and is finally resorbed in the late sub-adult stage, resulting in a thin cortex and a large infilled medullary cavity. The predominant bone tissues are parallel-fibred and lamellar-zonal for the forelimbs and hind limbs respectively. The oldest individual displayed an External Fundamental System indicating that the growth rate had decreased substantially by this stage. Variability is prevalent between the forelimb and hind limb as well as between early and late sub-adults Forelimb elements exhibit characteristics such as faster growing parallel-fibered bone tissue, slightly higher vascularization and a predominance of annuli over Lines of Arrested Growth (LAG) compared to the hind limb which exhibits poorly vascularized, slower growing lamellar-zonal bone interrupted by LAGs. These differences indicate that the forelimb grew more rapidly than the hind limb, possibly due to the method of locomotion seen in terrestrial species. The extensive bone resorption that occurs from the early sub-adult stage destroys much of the primary cortex and results in a significantly different ratio of inner and outer bone diameter (*p* = 3.59 × 10­^−5^; df = 28.04) as well as compactness (*p* = 2.91 × 10­^−5^; df = 31.27) between early and late sub-adults. The extensive bone resorption seen also destroys the ecological signal and infers an aquatic lifestyle for this species despite it being clearly terrestrial. This supports the results of other studies that have found that using bone microanatomy to determine lifestyle in testudines does not produce accurate results.

## Introduction

Testudines (Chelonia) are one of the oldest living groups of reptiles. The clade comprises three subgroups, namely tortoises, terrapins and turtles (Schmidt & Inger, 1957) and all, to some extent, have a protective encasing, formed from interlocking plates that act as armor (Rose, 1950). The origins and phylogenetic relationships of turtles has been debated for over four decades with procolophonid reptiles (Reisz & Laurin, 1991), sauropterygians (Rieppel & Debraga, 1996), pareisaurs (Lee, 1997), Lepidosauromorpha (Hill, 2005; Liu et al., 2011), archosaurs (Crawford et al., 2012, 2015; Field et al., 2014; Wang et al., 2013) and *Eunotosaurus* (Lee, 2013) being proposed as the ancestors of turtles. Furthermore, the use of morphological and molecular techniques have resulted in different hypotheses regarding the relationships of extant testudines (Evers & Benson, 2019), further complicating their origin and phylogenetic relationships.

Several researchers have focused on the testudine bone microanatomy, which provides information on life habits (Germain & Laurin, 2005; Kriloff et al., 2008; Canoville & Laurin, 2009; Laurin, Canoville & Germain, 2011). Given that the group includes a wide variety of lifestyles namely marine, freshwater, terrestrial and fossorial species, and that a fossorial origin of the turtle shell has been proposed (Lyson et al., 2016), understanding the link between function and bone microanatomy in this group is important for understanding their evolution. Animals with a pelagic lifestyle (e.g., dolphins, whales) have osteoporotic bones that comprise mostly spongy bone with a gradual transition from the medullary cavity to outer cortex. The medullary cavity is generally completely infilled by bone trabeculae (spongy bone) that is thought to improve diving capabilities by decreasing the weight of the bones (De Buffrénil & Schoevaert, 1988; De Ricqlès & De Buffrénil, 2001; Nakajima & Endo, 2013; Nakajima, Hirayama & Endo, 2014). In contrast, species that live in shallow water (e.g., manatees) tend to have pachyostotic bones that are highly compact and have no or tiny medullary cavities (Houssaye, 2009; Nakajima & Endo, 2013; Nakajima, Hirayama & Endo, 2014). These features are thought to aid in counter-acting buoyancy while in the water. Fossorial species may have thick bone walls (Laurin, Canoville & Germain, 2011; Montoya-Sanhueza & Chinsamy, 2017; Legendre & Botha-Brink, 2018; Heck et al., 2019) whereas terrestrial animals tend to have thinner cortices as well as clear medullary cavities and a sharp transition from medullary cavity to cortex compared to both aquatic or fossorial species.

Testudines do not always follow these patterns, but instead, contradict the pattern found in other amniotes (Germain & Laurin, 2005; Kriloff et al., 2008; Canoville & Laurin, 2010; Laurin, Canoville & Germain, 2011). Freshwater aquatic testudines tend to exhibit more compact bone and marine aquatic species trabecular infilling of the medullary cavity (in some cases to the extent where compact cortical bone is almost absent) whereas terrestrial species have smaller medullary cavities with a large proportion of compact cortical bone (Laurin, Canoville & Germain, 2011). Laurin and colleagues standardized their method by comparing the mid-diaphysis of the limb bones of sub-adult or fully grown individuals (Germain & Laurin, 2005). This region is generally the most appropriate as it should exhibit the strongest ecological signal because it preserves the thickest compact cortex and least secondary remodeling. A potential problem with this method is that the mid-diaphysis is assumed to represent the thickest part of the mid-shaft and this may not always be the case. In a study on the compactness of turtle humeri, Nakajima, Hirayama & Endo (2014) showed that the growth center (the point from where bone growth originates) and thus, thickest compact cortex, is not necessarily located in the mid-point of the diaphysis in testudines. The growth center shifts as the organism develops. Moreover, testudines experience distinct limb bone loading during terrestrial locomotion due to the orientation of bending and a high degree of torsion (twisting) (Butcher & Blob, 2008). Limb bone loads might be expected to be low in terrestrial testudines because of their slow walking speed (Walker, 1971), but as noted in Butcher & Blob (2008) the robust nature of the limb bones, might be a result of limb bone stress and its association with the sprawling orientation seen in terrestrial testudine locomotion. The most noteworthy feature of terrestrial testudine limb bones is the oddly shaped humerus, which experiences a remarkably high degree of torsion. This feature changes the mechanical property of the bone and influences the microanatomy of the element, thus potentially affecting data used to predict lifestyle.

*Stigmochelys pardalis* is a large terrestrial tortoise from South Africa (Drabik-Hamshare & Downs, 2017). Both sexes may dig shallow scrapes for shelter during periods of inactivity while females also dig scrapes during egg laying (Alexander & Marais, 2007). However, it is not considered to be fossorial as digging is limited to short periods and the shallow scrapes are more similar to scratch digging than burrow making. *S. pardalis* is a relatively common species, which allowed us to obtain hatchling, juvenile, sub-adult and adult specimens that all died from natural causes. To date, no study has tested the viability of using the mid-diaphysis to assess lifestyle in terrestrial testudines, nor the effect of ontogeny on the microanatomy of the limb bones. Here, we describe the osteohistology and bone microanatomy in an ontogenetic series of the leopard tortoise *Stigmochelys pardalis*. These samples were used for the aim of assessing ontogenetic and inter-elemental variation in the osteohistology and bone microanatomy of this species.

## Methods

Limb bones (61 samples) belonging to 38 individuals and four age classes (Juvenile: 19 samples; Early sub-adult: 13 samples; Late sub-adult: 28 samples; Adult: 1 sample) were used for the analysis of ontogenetic and inter-elemental osteohistological variability in the leopard tortoise *S. pardalis*. Museum specimens (MVD-R) were donated to the National Museum, Bloemfontein between 2000 and 2015. Samples were collected by farmers on agricultural lands in the Free State and Northern Cape provinces, South Africa. The bones were degreased in a liquid soap solution for approximately 2 weeks in direct sunlight to remove any soft tissue. Bones were labelled according to the Karoo Vertebrate Paleontology Modern Vertebrate Database of Reptiles (MVD-R) at the National Museum, Bloemfontein, where the specimens are stored. Multiple elements belonging to the same individual were given the same number (e.g., MVD-R 12) but different codes (e.g., right humerus: MVD-R12b; left humerus: MVD-R 12a; right radius: MVD-R 12c). Furthermore, an adult humerus (MNHN-ZA-AC 2010-2), previously used in Nakajima, Hirayama & Endo (2014), was later obtained and used in this study. Due to difficulties in obtaining naturally deceased adult specimens and the destructive nature of thin sectioning, only one adult specimen was obtained and utilized for the project. All samples were measured and divided into the following categories: juvenile, 0–20% of the maximum known size; early sub-adult, 21–49% of maximum size; late sub-adult, 50–79% of the maximum size; adult, 80–100% of the maximum size (Table 1). For brevity, percentage of the maximum known size for each element is referred to as % adult because humerus (MNHN-ZA-AC 2010-2) is considered to be 100% adult (reference for 100% adult size). Multiple elements of the same individual made it possible to calculate percentage adult between elements.

**Table 1:** Anatomical and ontogenetic information pertaining to sampled specimens.

Element	Accession #	Age class	Limb side	Individual	Bone length (mm)	Midshaft diameter (mm)	% Adult	Sectioning plane
Humerus	MVD-R-12b	J	R	1	8.1	1	5.0	Diaphysis
	MVD-R-12a	J	L	1	8.2	1	5.0	Diaphysis
	MVD-R-11b	J	R	2	9.5	1.2	5.8	Diaphysis
	MVD-R-11a	J	L	2	9.6	1.2	5.9	Diaphysis
	MVD-R-10a	ESA	L	3	45.9	8.6	28.2	Diaphysis
	MVD-R-9	ESA	R	4	60.4	7.3	37.1	Diaphysis
	MVD-R-8	ESA	R	5	60.9	6.2	37.4	Diaphysis
	MVD-R-7	ESA	R	6	68.8	7.8	42.3	Diaphysis
	MVD-R-6	ESA	R	7	73.4	8.6	45.1	Diaphysis
	MVD-R-5	LSA	R	8	81.2	9.6	49.9	Diaphysis
	MVD-R-4	LSA	L	9	81.4	9.8	50.0	Diaphysis
	MVD-R-3	LSA	R	10	87.3	10.3	53.6	Diaphysis
	MVD-R-2a	LSA	L	11	94.5	11.6	58.0	Diaphysis
	MVD-R-1	LSA	R	12	97.9	10.3	60.1	Diaphysis
	MNHN-ZA-AC 2010-2	A	R	38	162.8	23.5	100.0	Diaphysis
Radius	MVD-R-12c	J	R	1	4.9	0.4	3.0	Metaphysis
	MVD-R-18a	J	R	13	12.2	0.9	7.5	Diaphysis
	MVD-R-17	ESA	R	14	42.4	48	26.0	Diaphysis
	MVD-R-16	LSA	L	15	50.1	5	30.8	Diaphysis
	MVD-R-15	LSA	L	16	51.7	5.6	31.8	Diaphysis
	MVD-R-14	LSA	L	17	52.5	6.4	32.2	Diaphysis
	MVD-R-13	LSA	R	18	54.3	6.1	33.4	Diaphysis
	MVD-R-2b	LSA	R	11	56.3	6.4	34.6	Diaphysis
Ulna	MVD-R-12e	J	R	1	4.7	4.4	2.9	Metaphysis
	MVD-R-12d	J	L	1	4.8	4.7	2.9	Diaphysis
	MVD-R-18b	J	R	13	12.5	11.8	7.7	Diaphysis
	MVD-R-10b	ESA	L	3	31.8	30	19.5	Diaphysis
	MVD-R-23	ESA	L	19	39.8	37.5	24.4	Diaphysis
	MVD-R-22	ESA	R	20	47.8	45	29.4	Diaphysis
	MVD-R-21	LSA	L	21	53.9	50.8	33.1	Diaphysis
	MVD-R-20	LSA	R	22	55.2	52	33.9	Diaphysis
	MVD-R-19	LSA	L	23	62.7	59.1	38.5	Diaphysis
	MVD-R-2c	LSA	R	11	64.5	60.8	39.6	Diaphysis
Femur	MVD-R-12g	J	*R*	1	7.2	5.1	4.4	Diaphysis
	MVD-R-12f	J	L	1	7.4	5.2	4.5	Metaphysis
	MVD-R-11c	J	L	2	11.5	8.1	7.1	Diaphysis
	MVD-R-10c	ESA	L	3	41.5	29.2	25.5	Diaphysis
	MVD-R-28	ESA	R	24	49.2	34.6	30.2	Diaphysis
	MVD-R-27	LSA	R	25	73.6	51.8	45.2	Diaphysis
	MVD-R-26	LSA	L	26	73.9	52	45.4	Diaphysis
	MVD-R-25	LSA	R	27	84.3	59.4	51.8	Diaphysis
	MVD-R-24	LSA	R	28	85.8	60.5	52.7	Diaphysis
	MVD-R-2d	LSA	R	11	86.3	60.8	53.0	Diaphysis
Tibia	MVD-R-12i	J	R	1	6.2	6	3.8	Metaphysis
	MVD-R-12h	J	L	1	6.2	6.1	3.8	Diaphysis
	MVD-R-11d	J	L	2	8.91	8.73	5.5	Metaphysis
	MVD-R-11e	J	R	2	8.3	8.1	5.1	Diaphysis
	MVD-R-33	ESA	L	29	43.8	42.9	26.9	Diaphysis
	MVD-R-32	LSA	R	30	53.3	52.3	32.7	Diaphysis
	MVD-R-31a	LSA	L	31	56.3	55.1	34.6	Diaphysis
	MVD-R-30	LSA	R	32	56.6	55.5	34.8	Diaphysis
	MVD-R-2e	LSA	L	11	62	60.8	38.1	Diaphysis
	MVD-R-29	LSA	L	33	63.4	62.1	38.9	Diaphysis
Fibula	MVD-R-12k	J	L	1	6	5.8	3.7	Diaphysis
	MVD-R-12j	J	R	1	6.1	5.9	3.7	Metaphysis
	MVD-R-11f	J	L	2	7.8	7.5	4.8	Diaphysis
	MVD-R-36	ESA	R	34	46.7	44.9	28.7	Diaphysis
	MVD-R-31b	LSA	L	35	54.3	52.2	33.4	Diaphysis
	MVD-R-35	LSA	R	36	55.5	53.4	34.1	Diaphysis
	MVD-R-34	LSA	L	37	62	59.6	38.1	Diaphysis
	MVD-R-2f	LSA	L	11	63.1	60.8	38.8	Diaphysis

**Note:**

Abbreviations: MVD-R, Modern Vertebrate Database Reptiles; R, Right; L, Left; J, Juvenile; ESA, Early sub-adult; LSA, Late sub-adult; A, Adult.

Bones were embedded in a Struers epoxy resin and cut into 1.5 mm sections using an Accutom-50 thin sectioning machine. Sections were glued to frosted slides using an Epolam 2022 industrial glue and placed into a vacuum chamber for 10 min to remove any excess bubbles. Specimens were then ground to approximately 150 µm using a diamond tipped grinding cup–wheel in the Accutom-50 machine and then manually sanded down using a fine sand-paper and polished using a Struers LaboPol-5 polishing machine.

Sections were viewed using a Nikon DS-FI1 polarizing petrographic microscope. Magnification images (4×) were rendered together (in normal and cross-polarized light with lambda compensator) to form images of whole cross-sections for the analysis of the microanatomy. Bone tissue images at 10× magnification were captured and were used to quantify the cortical vascularization. Images were captured using a DS-FI1 digital camera mounted on the microscope and saved using the computer program, NIS-Elements D 3.

Global compactness (Cg) was quantified to determine cortical and trabecular changes between ontogenetic stages as a result of cortical resorption. This was done by comparing the proportion of compact vs spongy bone at the middle of the diaphysis of each bone. Vascular canals were not included in the determination of Cg because their inclusion yielded no significant change to the global value. Sections were converted into binary black and white images using Corel Photo Paint X4. The extent of compactness was then determined using Bone Profiler for Windows V4.5.8 (Girondot & Laurin, 2003) and further information regarding Min (value for the minimum compactness), Max (value for the maximum compactness), *S* (the value proportional to the width of the transition zone between the medullary cavity and cortex) and *P* (the value of the transition area between the medullary cavity and compact cortex) were also obtained. These values were used to predict lifestyle for the humerus (Canoville & Laurin, 2010), radius (Laurin, Canoville & Germain, 2011) and tibia (Kriloff et al., 2008). The lifestyle readings of the ulnae and fibulae were not determined due to a lack of software and the lifestyle inference of the femora (Quemeneur, De Buffrénil & Laurin, 2013) was not performed because certain variables (Ti: snout length and Rmin) used in the determination of lifestyle could not be obtained. The *R*/*t* value, where *R* is the outer radius of the bone and *t*, the thickness of the cortical wall, was also recorded as this provides an indication of the thickness of the cortical bone wall (Currey & Alexander, 1985). These values were converted to *K* (the ratio of the internal diameter to the outer diameter of the bone) according to the formula outlined in Currey & Alexander (1985) in order to allow for comparisons with the literature. *K* values and Cg were also used to test for differences between early and late sub-adults. The package “lmerTest” of the R software (3.6.1 for Windows) was used to perform linear mixed effects models with age class as the fixed effect, and individual number and element type as random effects.

The degree of vascularization was calculated in order to compare vascularization quantitatively between elements and with the literature. Images were analyzed in Image J 1.50i where the perimeter of all the vascular canals were traced, the area occupied by the canals was calculated and then divided by the total area of the cortex. The area occupied by the canals was expressed as a percentage. The identification and analysis of growth marks was done using transverse sections in normal and cross polarized light at both 4× and 10× magnifications. Sections were analyzed for: (1) Lines of Arrested Growth (LAG), a temporary but complete cessation of growth; (2) Annuli, the substantial temporary decrease in growth rate and (3) External Fundamental System (EFS), the rapid and successive deposition of growth marks at the periphery of the cortex. Additionally, Sharpey’s fibers were also identified using 10× normal and cross polarized light images.

## Results

### Humeri

**Figure 1:**
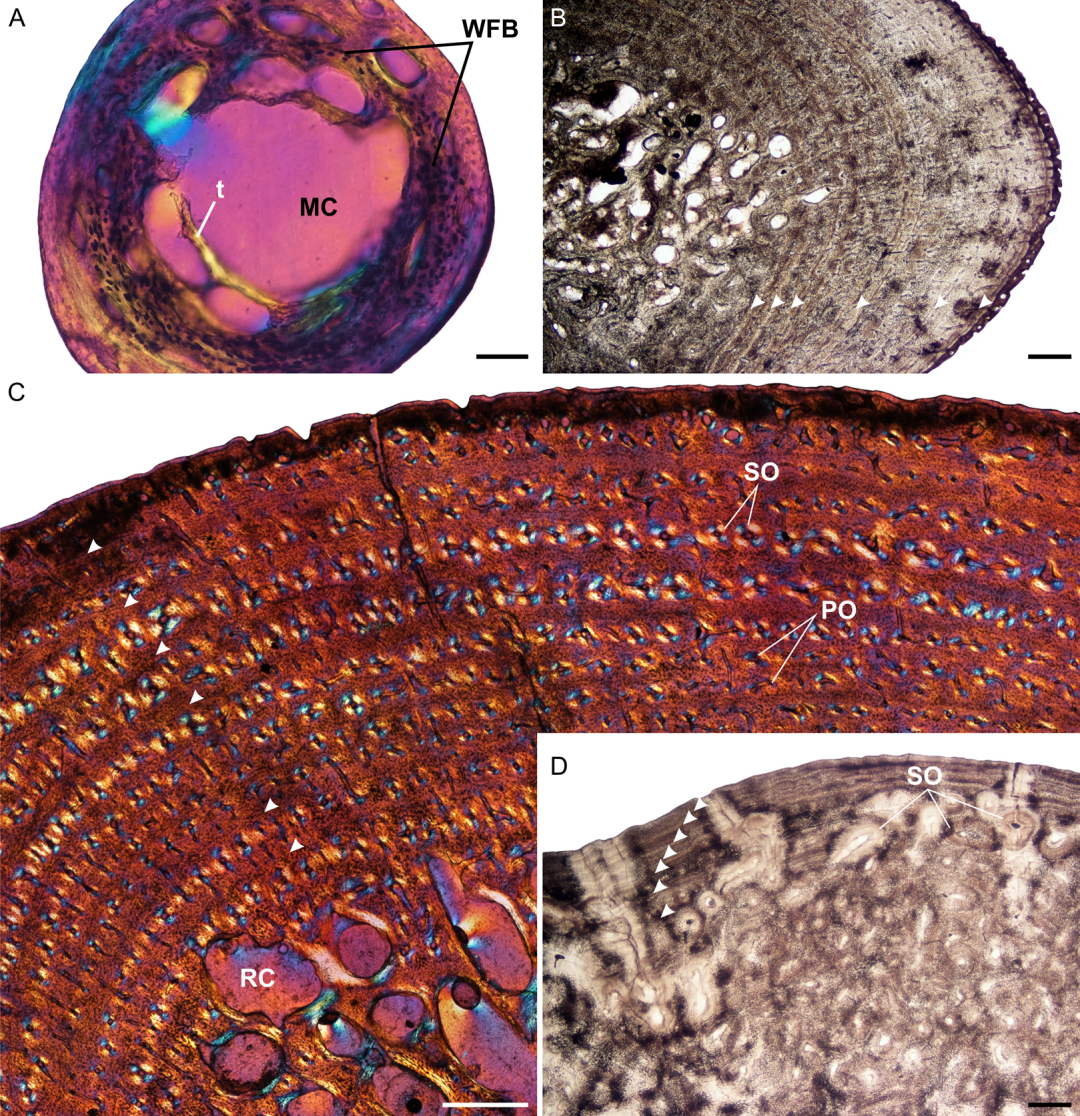
Humeral osteohistology of *Stigmochelys pardalis*. (A) Whole cross-section of juvenile MVD-R-12a showing a relatively thin cortex of woven-fibred bone. (B) High magnification of early sub-adult MVD-R-7 showing parallel-fibered bone interrupted by growth marks. The uneven bone periphery shows that the bone was still actively growing at the time of death. (C) Late sub-adult MVD-R-5 showing circumferential rows of primary osteons in parallel-fibered bone. Arrowheads indicate annuli. (D) Adult MNHN-ZA-AC-2010-2 showing a secondarily remodeled cortex and seven closely spaced LAGs (arrowheads) indicating an EFS. Abbreviations: MC, medullary cavity; PFB, parallel-fibered bone; PO, primary osteons; RC, resorption cavity; SO, secondary osteons; t, trabecular bone; WFB, woven-fibred bone. Scale bars equal 100 μm in (A) and 500 μm in (B), (C) and (D).

Juveniles have large, centrally placed almost, if not completely, open medullary cavities (*K* = 0.6). The cavities become increasingly infilled with bony trabeculae from the early sub-adult stage resulting in a gradual transition zone from spongy to compact bone (Fig. 1A) resulting in an average Cg of 0.733. Early sub-adults have thicker compact cortices (average *K* = 0.26) with a Cg over 0.93, which is an indication of their small medullary cavities and thick cortices. Cortical drift is prominent in early sub-adults as the medullary cavities tend to be situated on the posterior side of the bone. As the bones undergo more torsion with age, the placement of the medullary cavity becomes increasingly asymmetrical. Late sub-adult specimens show a clear increase in the size of the medullary cavities which tend to be more centrally placed. Secondary osteons are absent from the juveniles, but older individuals have extensively resorbed areas surrounding the medullary cavity. The cortices of the late sub-adults have undergone extensive resorption and as a result, the compact cortices are thinner than the early sub-adults (average *K* = 0.49), particularly in the oldest individual. However, the extensive bony trabeculae in the medullary cavities still results in a high compactness in all late sub-adult specimens. Due to the retention of various characteristics (smaller medullary cavities and thicker cortices), late sub-adult specimens (MVD-R-4 and MVD-R-5) have a higher Cg (0.917 and 0.914 respectively), than the earlier sub-adults. In contrast, older individuals, have a lower average Cg value of 0.836, which is expected considering the extensive resorption of primary cortical bone seen in these specimens. The resorption cavities in older individuals extend into the mid-cortex, leaving very little of the primary compact cortex. Secondary osteons are concentrated in the peri-medullary region. Older sub-adults have less secondary osteons overall, but secondary osteons are larger and more prominent compared to younger sub-adults. Adult specimen MNHN-ZA-AC 2010-2 has an average compactness of 0.87, lower than the oldest late sub-adult individual (MVD-R-1, *K* = 0.91). The difference between their compactness may be due to the larger medullary cavity and the extensive amount of secondary remodeling and resorption that has taken place. The cortex is scattered with randomly distributed secondary osteons and cavities of various sizes extend from the medullary cavity to the periphery of the bone, which was seldom seen in any of the late sub-adults.

The juvenile humeri contain numerous, large, globular, randomly distributed osteocyte lacunae. The original collagen fibers cannot be detected making it difficult to classify the bone matrix. However, given the size, shape and abundance of osteocytes, the bone matrix is most likely woven-fibered (Fig. 1A). This is typical of embryos and juveniles (Francillon-Vieillot et al., 1990). Primary osteons had yet to develop but the cortical cavities are large resulting in an average vascularization of 4.75% (for left and right elements). Early sub-adults (Fig. 1B) have a predominantly parallel-fibered bone matrix. They contain many flattened and randomly distributed osteocyte lacunae, but clusters of larger circular lacunae were also observed in the inner cortex. Growth marks first appear in this age class, ranging from two single annuli to four single annuli and a double LAG (at the periphery). The type and pattern of vascular canals differed between specimens, ranging from predominantly simple canals, predominantly longitudinally-oriented primary osteons to a mixture of simple canals and longitudinally-oriented primary osteons. Vascularization decreases with age ranging from 0.24% in MVD-R-8 to 0.09% in MVD-R-7. MVD-R10a differs substantially from the rest of the early sub-adults as it contains many branched vascular canals forming a sub-plexiform arrangement. The surface on the anterior side of the bone is also uneven with clear active bone growth in this region (with some short radiating canals in this area as well).

The bone tissues of late sub-adults are mostly parallel-fibered with a mixture of flattened and oval osteocyte lacunae distributed throughout the cortex. However, MVD-R-5 has a lamellar-zonal bone matrix with organized flattened osteocyte lacunae throughout most of the section, but a parallel-fibered bone matrix with round osteocyte lacunae was observed in the inner-cortex. The bone tissues of MVD-R-3 and MVD-R-1 are predominantly parallel-fibered with clear lamellar zonal bone at the periphery of the bone. Late sub-adults are similar to the early sub-adults in that they also contain longitudinally-oriented primary osteons in circular rows (Fig. 1C). However, the less developed late sub-adults have more rows as less of the primary cortex has been resorbed. The cortices are very poorly vascularized with an average of 0.16%. Simple canals are mostly small longitudinally-oriented unbranched and with a number of canals exhibiting short anastomoses. Vascularization decreases substantially towards the periphery in some parts of the bones indicating a decrease in growth rate. A transition from parallel-fibered to lamellar-zonal bone can be seen in late sub-adult MVD-R3, indicating an overall decrease in growth rate. Numerous closely spaced growth marks are present in the outer cortex, which may represent an EFS, indicating skeletal maturity. Much of the primary bone in the inner to mid-cortex of adult specimen MNHN-ZA-AC 2010-2 has been destroyed by secondary reconstruction (Fig. 1D). However, there is a small patch of parallel-fibered bone with longitudinally-oriented primary osteons in the mid-cortex. Beyond this, the rest of the primary compact bone comprises lamellar-zonal bone with numerous LAGs (Fig. 1D). The outer cortex looks similar to MVD-R-3 with numerous closely spaced LAGs traversing the bone tissue, which likely represents an EFS.

Evidence of muscle insertions is visible by the presence of Sharpey’s fibers in early sub-adult MVD-R-8 on the dorsal side of the cortex. They were observed in late sub-adult MVD-R-1, indicating the attachment of muscles on the anterodorsal side of the bone (Table 2).

**Table 2:** Osteohistological information related to growth and muscle attachment in the *Stigmochelys pardalis* study sample.

Element	Accession #	Age class	%Vascularization	Growth marks	Evidence of attachment
Humerus	MVD-R-12b	J	4.9	–	–
	MVD-R-12a	J	7.2	–	–
	MVD-R-11b	J	3.5	–	–
	MVD-R-11a	J	3.7	–	–
	MVD-R-10a	ESA	0.2	3 Single annuli	–
	MVD-R-9	ESA	0.2	3 Single annuli	–
	MVD-R-8	ESA	0.2	4 Single annuli	Sharpey’s fibers
	MVD-R-7	ESA	0.1	1 Single lag, 4 single annuli	–
	MVD-R-6	ESA	0.1	2 Annuli	–
	MVD-R-5	LSA	0.3	9 Single annuli, 1 single LAG	–
	MVD-R-4	LSA	0.2	5 Annuli	–
	MVD-R-3	LSA	0.1	5 Single LAGs	–
	MVD-R-2a	LSA	0.2	5 Single annuli	–
	MVD-R-1	LSA	0.1	6 Single LAGs	Sharpey’s fibers
	MNHN-ZA-AC 2010-2 (xs 13)	A	–	7 Closely spaced single LAGs (EFS)	Sharpey’s fibers
Radius	MVD-R-12c	J	–	–	–
	MVD-R-18a	J	0.4	–	–
	MVD-R-17	ESA	0.7	5 Single LAGS	Sharpey’s fibers
	MVD-R-16	LSA	0.1	5 Single LAGS	Sharpey’s fibers
	MVD-R-15	LSA	0.2	5 Single annuli, single LAG	Sharpey’s fibers
	MVD-R14	LSA	0.1	4 Single LAGs	–
	MVD-R-13	LSA	0.1	3 Single LAGs	–
	MVD-R-2b	LSA	0.1	1 Single LAG,1 double annuli and 2 single annuli	Sharpey’s fibers
Ulna	MVD-R-12e	J	2.7	–	–
	MVD-R-12d	J	2.5	–	–
	MVD-R-18b	J	0.4	–	–
	MVD-R-10b	ESA	0.2	1 Single annuli	Sharpey’s fibers
	MVD-R-23	ESA	0.1	5 Single annuli	Sharpey’s fibers
	MVD-R-22	ESA	0.2	3 Single annuli, 2 single LAGs	–
	MVD-R-21	LSA	0.1	2 Single LAGs	Sharpey’s fibers
	MVD-R-20	LSA	0.1	7 Single annuli	Sharpey’s fibers
	MVD-R-19	LSA	0.1	3 Single LAGs, 1 double lag,	Sharpey’s fibers
	MVD-R-2c	LSA	4.5	2 Single annuli, 1 single LAG	Sharpey’s fibers
Femur	MVD-R-12g	J	2	–	–
	MVD-R-12f	J	4.8	–	–
	MVD-R-11c	J	0.3	Hatchling line	Sharpey’s fibers
	MVD-R-10c	ESA	0.1	2 Single LAGs	Sharpey’s fibers
	MVD-R-28	ESA	0.1	6 Double LAGs	Sharpey’s fibers
	MVD-R-27	LSA	0.1	2 Single LAGs, 1 double LAG	–
	MVD-R-26	LSA	0.1	4 Single annuli	Sharpey’s fibers
	MVD-R-25	LSA	0.1	1 Double LAG, 2 single LAGs	Sharpey’s fibers
	MVD-R-24	LSA	0.1	4 Single LAGs, 2 double LAGs	–
	MVD-R-2d	LSA	0.1	2 Single LAGs	–
Tibia	MVD-R-12i	J	0.8	–	–
	MVD-R-12h	J	3.1	–	–
	MVD-R-11e	J	1.6	–	–
	MVD-R-11d	J	0.5	–	–
	MVD-R-33	ESA	0.1	3 Single LAGs	–
	MVD-R-32	LSA	0.1	5 Single annuli	Sharpey’s fibers
	MVD-R-31a	LSA	0.1	Triple annulus, 2 single annuli and 2 double LAGs	Sharpey’s fibers
	MVD-R-30	LSA	0.1	Double annuli, 1 single LAG, and 2 double LAGs	Sharpey’s fibers
	MVD-R-2e	LSA	0.1	1 Double LAG, 5 single LAGs	–
	MVD-R-29	LSA	0.1	6 Single LAGs	Sharpey’s fibers
Fibula	MVD-R-12k	J	0.9	–	–
	MVD-R-12j	J	–	–	–
	MVD-R-11f	J	–	–	Sharpey’s fibers
	MVD-R-36	ESA	0.1	6 Single LAGs	–
	MVD-R-31b	LSA	0.1	Double annuli, single LAG and double LAG	Sharpey’s fibers
	MVD-R-35	LSA	0.1	8–10 Single LAGs	Sharpey’s fibers
	MVD-R-34	LSA	0.1	4 Single LAGs	Sharpey’s fibers
	MVD-R-2f	LSA	0.1	8 Single LAGs	Sharpey’s fibers

**Note:**

Abbreviations: MVD-R, Modern Vertebrate Database Reptiles; LAG, Line of arrested growth; EFS, External fundamental system.

### Radii

**Figure 2:**
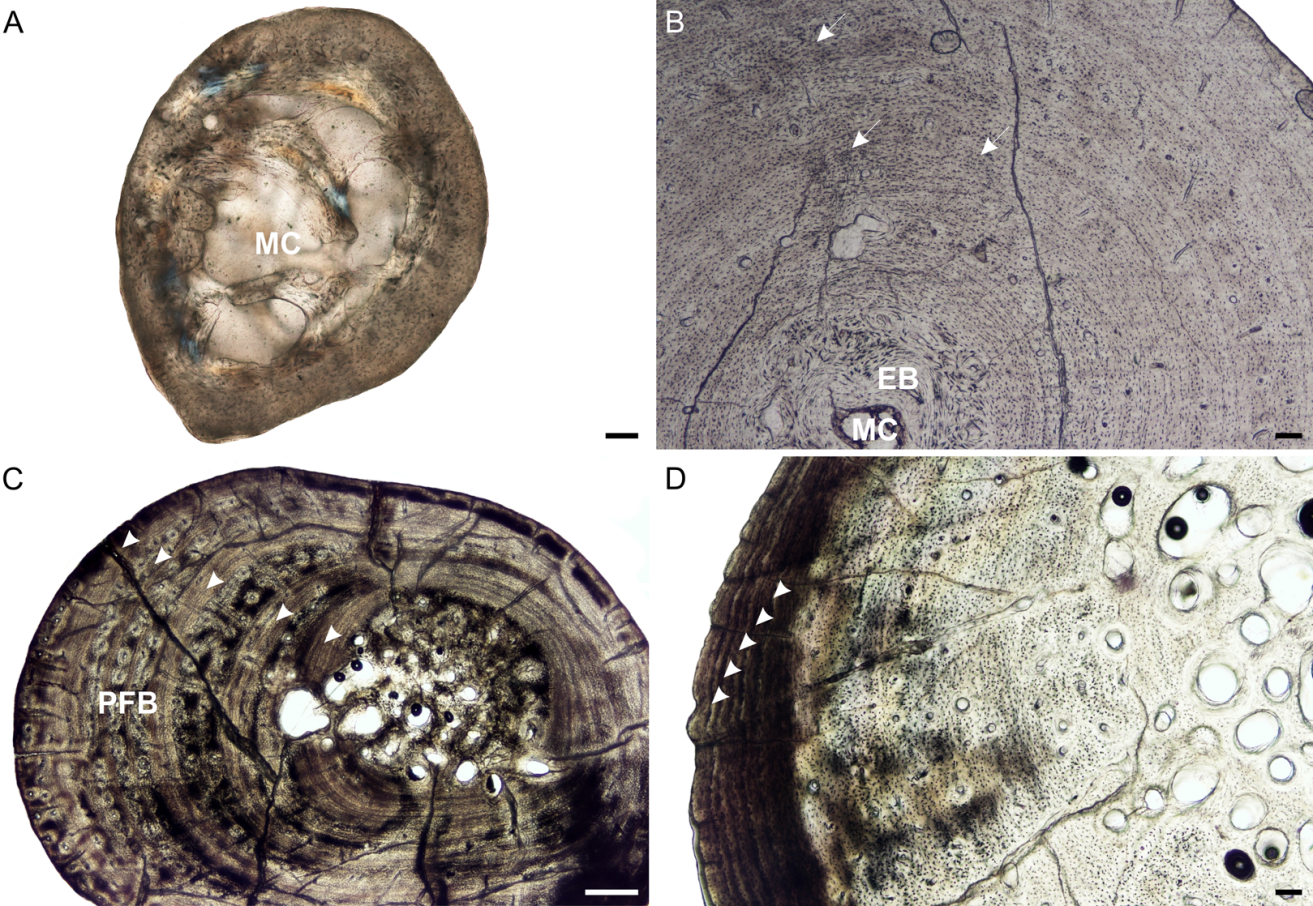
Radial osteohistology of *Stigmochelys pardalis*. (A) Whole cross-section of juvenile MVD-R-18a showing a few trabeculae traversing the medullary cavity. (B) Early sub-adult MVD-R-17 showing a tiny medullary cavity surrounded by a thick region of endosteal bone and a thick cortex. Arrows indicate Sharpey’s fibers. (C) Late sub-adult MVD-R-14 showing primary osteons in parallel-fibered bone interrupted by thick annuli (arrowheads). (D) Late sub-adult MVD-R-15 showing five closely spaced LAGs (arrowheads) at the bone periphery. Abbreviations: EB, endosteal bone; MC, medullary cavity; PFB, parallel-fibered bone. Scale bars equal 100 μm in (A), (B) and (C), and 500 μm in (D).

The juvenile radii contain a centrally placed oval medullary cavity with a few bony trabeculae traversing it (Fig. 2A). There are smaller cavities surrounding the large cavity where bone has yet to form. Juvenile MVD-R-18a has a high Cg (0.809) probably because the bony trabeculae traversing the medullary cavity are relatively thick, so increasing the overall compactness of the bone. The cortex also has relatively few cortical cavities. The Cg of juvenile MVD-R-12c is lower (0.424), but the section is from the metaphysis where the compact cortex is narrower. This is also noticeable in the different *K* values of MVD-R-12c (0.754) and MVD-R-18a (0.591). Only one early sub-adult radius was available for study, MVD-R-17. This bone has a tiny central area with four cavities just off-center (Fig. 2B). Early sub-adult MVD-R-17 shows clear changes in its cortical microstructure compared to the juveniles. The cortex is extremely thick (*K* = 0.036) and comprises most of the cross sectional area (Cg = 0.989). The medullary cavities of the late sub-adults are larger than younger individuals due to the primary bone tissue being more heavily resorbed. The transition zone from cancellous to compact bone is more gradual in the older late sub-adults resulting in a broad transition zone. Younger late sub-adults show similar characteristics to those of the early sub-adult and have an average Cg of 0.952. The older individuals have larger medullary cavities and as a result, thinner cortices but the average Cg remains high at 0.9. The late sub-adults, as a result of continued resorption and growth, have numerous randomly distributed secondary osteons and resorption cavities ranging in size from small to large. Secondary osteons are distributed throughout the cortex in between circular rows of primary osteons. Secondary remodeling is concentrated in the peri-medullary region and various parts of the inner cortex, but it does extend to the periphery in MVD-R-15 where a number of secondary osteons are situated in between the circular row of primary osteons and scattered towards the periphery of the bone.

It is difficult to observe the orientation of the collagen fibers in the juveniles and therefore identifying the bone matrix is problematic. However, the presence of abundant, randomly distributed oval osteocyte lacunae is suggestive of a woven-fibered bone matrix, similar to the juvenile humeri. There are also patches of more organized osteocyte lacunae however, suggesting that the bone comprises a mixture of woven and parallel-fibered bone. Primary osteons are absent and vascularization is low with an average of 0.42%. The early sub-adult reveals an inner cortex of parallel-fibered bone, but becomes more slowly forming lamellar-zonal bone from the mid-cortex where the osteocyte lacunae become flattened and highly organized. The bone tissue contains a few small, longitudinally-oriented simple canals and primary osteons distributed throughout the cortex (vascularization 0.65%). There is a clear decrease in vascularization towards the periphery, indicating a decrease in growth rate. The bones of the late sub-adults contain a mixture of randomly distributed oval osteocyte lacunae and more organized, flattened osteocyte lacunae. MVD-R-15 has numerous primary osteons that are randomly distributed, but in some cases, arranged in circular rows in the inner and mid-cortex. Very few primary osteons are present in the other late sub-adults (Fig. 2C), which mostly contain longitudinally-oriented simple vascular canals resulting in an average vascularization of 0.11%. The bone tissues are mostly parallel fibered (Fig. 2C), but some specimens exhibit the more slowly forming lamellar-zonal bone. The youngest individual of this age class (MVD-R-16) exhibits lamellar-zonal bone throughout its cortex except in zones with longitudinally-oriented primary osteons in circular rows where thick parallel-fibered bone is present. Older individuals transition from parallel-fibered bone into lamellar-zonal bone in the outer cortex and retain this bone tissue to the periphery and together with a low vascularization, indicate a decrease in growth rate.

Annuli appear in the inner cortex of the early sub-adult with the appearance of, sometime double, LAGs towards the periphery. Numerous annuli and LAGs are present in all the late sub-adults (Fig. 2C). Four closely spaced LAGs were observed at the periphery of MVD-R-14, which likely represents an EFS and thus, skeletal maturity (Fig. 2D) (Table 2).

Prominent deep (almost to the mid-cortex) Sharpey’s fibers were observed in one region of the early sub-adult and were found in several late sub-adults (Table 2). MVD-R-2b and MVD-R-16 have shallow Sharpey’s fibers in the outer cortex and periphery whereas younger individual MVD-R-16 has deep Sharpey’s fibers extending into the inner cortex of the bone.

### Ulnae

**Figure 3:**
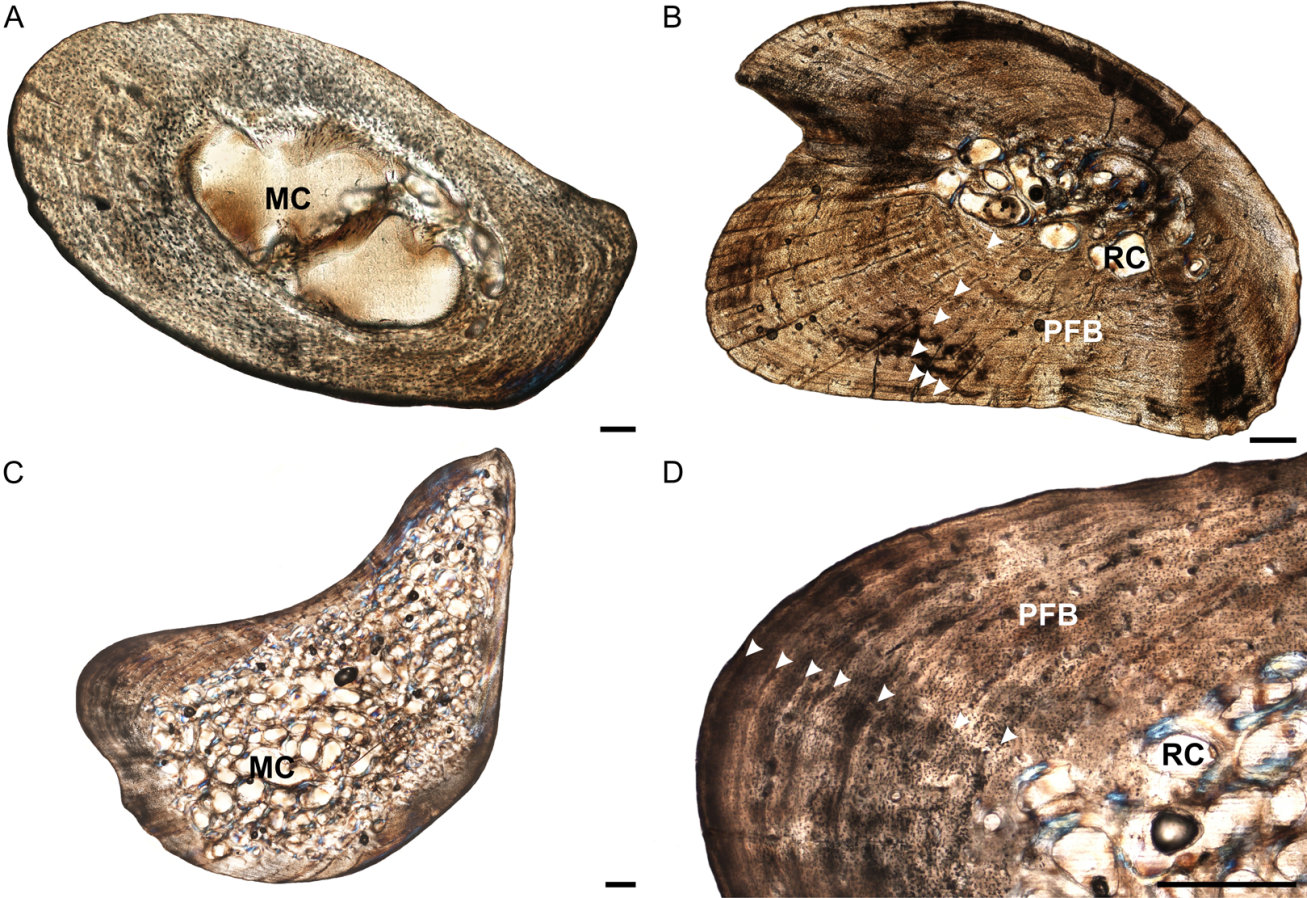
Ulna osteohistology of *Stigmochelys pardalis*. (A) Whole cross-section of juvenile MVD-R-18b showing a relatively thick cortex. Arrow indicates possible Sharpey’s fibers. (B) Early sub-adult MVD-R-22 showing an infilled medullary cavity surrounded by a thick cortex of parallel-fibered bone interrupted by annuli and LAGs (arrowheads). (C) Late sub-adult MVD-R-19 showing a “boomerang”-shaped bone with a medullary cavity that is completely infilled by trabeculae. (D) High magnification of late sub-adult MVD-R-19 showing parallel-fibered bone interrupted by annuli and LAGs (arrowheads). Abbreviations: MC, medullary cavity; PFB, parallel-fibered bone; RC, resorption cavity. Scale bars equal 100 μm in (A) and 500 μm in (B), (C) and (D).

Medullary cavities in the juveniles are essentially centrally placed and are large with either completely open medullary cavities or are traversed by one or two thick bony trabeculae (Fig. 3A). The cortex is relatively thin in the juveniles (average *K* = 0.55). The youngest individual (MVD-R-12e) has a relatively low Cg of 0.524 compared to older individuals but, the small size of the bone could result in a single section giving a different reading as it will represent the metaphysis of the bone. The oldest juvenile (MVD-R-18b) has a higher Cg of 0.834. Early sub-adults show a substantial decrease in the overall size of the medullary cavity, which is comprised of several small cavities (Fig. 3B). The medullary cavities of the early sub-adults are either circular in shape or resemble the shape of the ulna itself resulting in a “boomerang” shape. The early sub-adult stage is characterized by thicker cortices (average *K* = 0.16) and more bone resorption (in the form of resorption cavities and secondary osteons) in the peri-medullary region. These thicker cortices and absence of completely open medullary regions (compared to juveniles) results in a higher degree of compactness (average Cg of 0.939). The medullary cavities of the late sub-adults are circular “boomerang’’ shaped resembling the shape of the ulna itself (Fig. 3C). Some late sub-adults show a slight increase in the size of the medullary cavity, but are otherwise similar to the early sub-adults with regards to medullary cavity size, shape and placement. Larger late sub-adults have medullary cavities that occupy a larger percentage of the entire bone, leaving very little of the primary cortex and extensive trabecular infilling creating a broad transition zone to compact cortex. The size of the medullary cavity increases in the later developmental stages and as a result, a noticeable decrease in the thickness of the cortex as seen in the average *K* value of 0.67 compared to 0.16 in the early sub-adults. However, as a result of numerous bony trabeculae occupying the medullary cavity, Cg is still high (average Cg 0.883). Both early and late sub-adults have secondary osteons and resorption cavities in the peri-medullary region and inner cortex.

The bone tissues of the juveniles are similar to those seen in the humeri and radii. Osteocyte lacunae are generally evenly distributed throughout the bone matrix, but appear more random in the inner cortex and are arranged in parallel towards the outer cortex, suggesting an initial deposition of a woven-fibered bone matrix and then a transition to parallel-fibered bone. Vascularization is limited (average 1.54%). The bone matrix in the early sub-adults is predominantly parallel-fibered with some lamellar-zonal bone, similar to the radii. There are a few longitudinally-orientated primary osteons limited to the inner and mid-cortex, but most of the vascular canals are simple. In certain areas of the thickest part of the cortex radiating canals were observed, indicating more active growth in these regions. Vascularization decreases substantially towards the periphery and as a result, early sub-adults average 0.15%. In the late sub-adults the bone matrix is predominantly parallel-fibered with some specimens showing a transition to slower growing lamellar-zonal bone from the mid to outer cortices in regions where circular rows of primary osteons are present. The late sub-adults have small primary osteons and a few simple vascular canals resulting in a poor vascularization of 0.2% (Fig. 3D). Circular rows of longitudinally-oriented primary osteons are also present in these specimens, similar to that seen in the early sub-adults and other elements. Radiating canals in certain specimens, such as MVD-R-20, show a regional increase in bone growth and are found in areas where the cortex is thicker. Vascularization is generally consistent, but decreases substantially at the sub-periosteal surface.

The quantity of growth marks differed between specimens, most probably due to differences in bone resorption (Table 2). Sharpey’s fibers are present on the dorsal and ventral sides of the cortical bone. They are mainly limited to the periphery or shallow cortex in early sub-adults and extend further towards the medullary cavity in older late sub-adults.

### Femora

**Figure 4:**
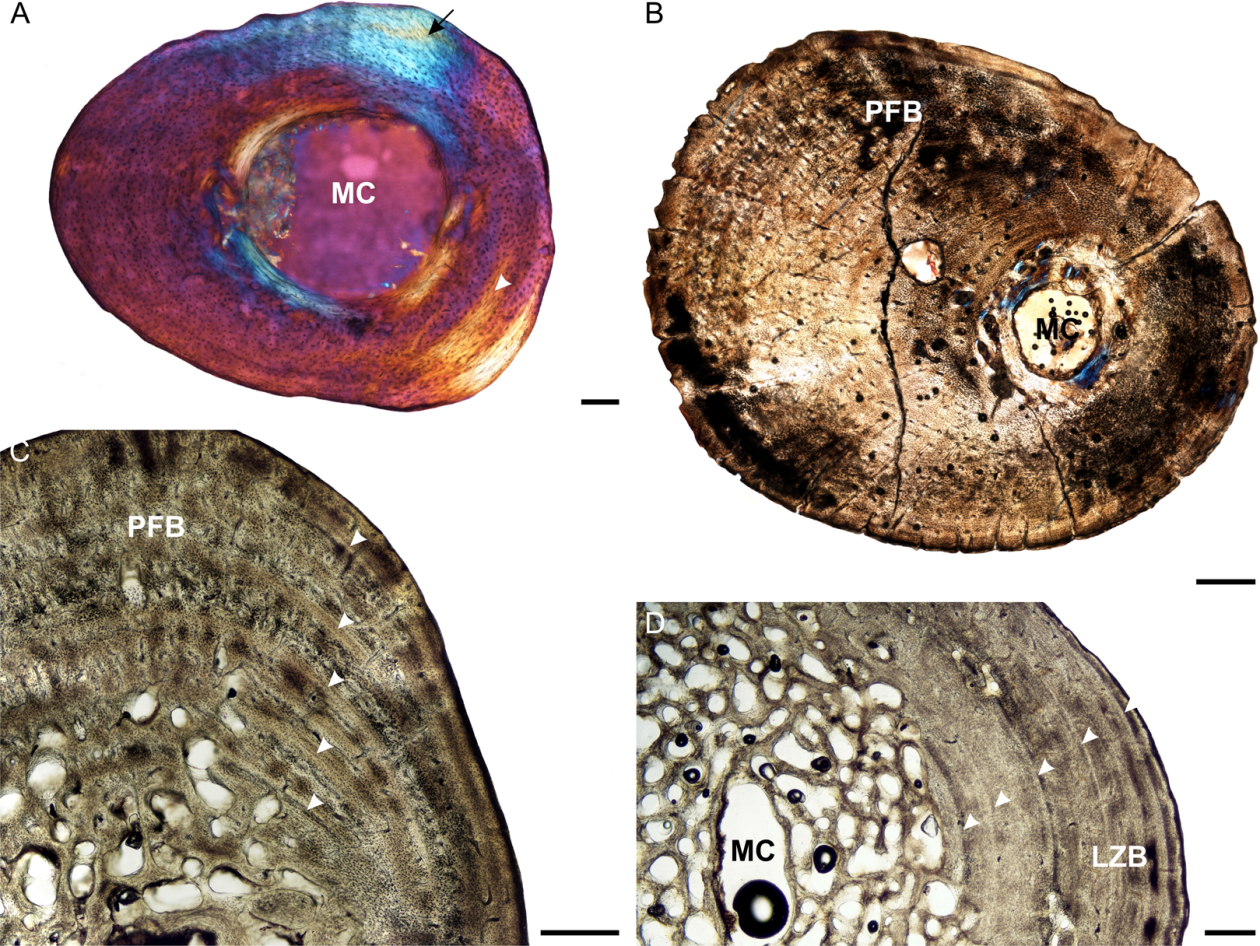
Femoral osteohistology of *Stigmochelys pardalis*. (A) Whole cross-section of juvenile MVD-R-11c in cross-polarized light showing a relatively thick cortex and a possible Sharpey’s fibers attachment site (black arrow). (B) Early sub-adult MVD-R-10c showing a tiny medullary cavity and very thick cortex of parallel-fibered bone. (C) Early sub-adult MVD-R-28 showing parallel-fibered bone interrupted by thick annuli (arrowheads). (D) High magnification of late sub-adult MVD-R-2d showing lamellar-zonal bone. Arrowheads indicate LAGs. Abbreviations: MC, medullary cavity; PFB, parallel-fibered bone. Scale bars equal 100 μm in (A) and 500 μm in (B), (C) and (D).

Juvenile specimens have circular, centrally placed medullary cavities, similar to the elements of the forelimb (Fig. 4A). MVD-R-12 and MVD-R-11c have open medullary cavities with no bony trabeculae. However, MVD-R-11c has smaller cavities surrounding the main opening. The cortices of the juveniles are moderately thick (average *K* = of 0.48) and have an average Cg of 0.71. The early sub-adults are limited to two specimens, MVD-R-10c and MVD-R-28, which both have tiny open medullary cavities at the center with smaller surrounding cavities that extend towards the ventral side of the bone (Figs. 4B and 4C). There is a relatively rapid transition from cancellous to compact bone. However, early sub-adult MVD-R-28 shows clear signs of resorption taking place in parts of the cortex that will eventually form part of the medullary cavity. The cortices of both early sub-adult specimens are thick (average *K* = 0.25) and the Cg is high, averaging 0.966. Secondary osteons appear in the early sub-adults, but are rare. Much of the primary cortex has been resorbed in the late sub-adults. Medullary cavities are infilled with fine bony trabeculae forming cavities throughout the medullary region. The medullary cavities are generally circular or oval in shape. The cortical regions of the late sub-adults are relatively thin (average *K* = 0.65) and the Cg is moderately high (average Cg 0.828). Secondary osteons are few and found mainly within the perimedullary region.

Osteocyte lacunae in the juveniles vary from flat to oval and are generally relatively large. A random distribution of osteocyte lacunae in MVD-R-12 suggests the presence of a woven-fibered bone matrix, similar to that seen in the other elements. However, the more organized nature of the osteocyte lacunae in MVD-R-11 suggests that the bone matrix is more parallel-fibered. Vascularization ranges from 0.29% to relatively high at 4.8%. Both early sub-adults exhibit parallel-fibered bone tissue (Figs. 4B and 4C). MVD-R-10c still shows signs of active growth as seen by the uneven, newly deposited bone surface (Fig. 4B). Vascularization is similar between specimens (0.08%) with a mixture of branched radiating and unbranched longitudinally-oriented simple canals as well as primary osteons. There is at least one circular row of longitudinally-orientated primary osteons in each specimen. However, because of the small size of the vascular canals, the vascularization in the early sub-adults is very low. The bone tissue is generally comprised of lamellar-zonal bone. MVD-R-26 differs from the other specimens in having a mixture of bone tissues comprising predominantly parallel-fibered bone with patches of woven-fibered bone as seen by clumps of osteocytes, possibly indicating static osteogenesis. However except in MVD-R 2d that displayed prominent lamellar zonal bone (Fig. 4D). MVD-R-26 appears to have grown more rapidly than the other late sub-adults, which is not unexpected given that it falls within the lower size range of this age class. Vascularization in the late sub-adults is generally extremely limited (average 0.1%) as a result of the small, sparse vascular canals. There are mainly unbranched canals, but in MVD-R-2d and MVD-R-24, there are a number of branched canals with short anastomoses. Primary osteons are small, longitudinally-oriented and randomly distributed throughout the cortex. However, MVD-R-26 and MVD-R-2d have circular rows of primary osteons within the cortex.

Growth marks are absent in the juveniles except for a possible faint annulus in the mid-cortex of MVD-R-11c. This individual is very young (approximately a few weeks old, based on body size). Thus, it either hatched during an unfavorable growing season and its growth slowed very soon after birth, or the feature represents a hatchling line. Hatchling lines (also known as neonatal lines) are deposited during birth and represent a particularly stressful time in an individual’s life (De Buffrénil & Castanet, 2000) and particularly slow growth (De Buffrénil, Houssaye & Bohme, 2008). They are generally in the form of a LAG that differs from annual LAGs in that it is more pronounced. The annulus in MVD-R-11c is indistinct and cannot be followed around the whole cortex, thus it is unclear at this stage if it does represent a hatchling line. The early sub-adults exhibit growth marks generally in the form of LAGs, but annuli were also observed in MVD-R-26, indicating that growth did not cease completely during the unfavorable growing season. This further supports the suggestion that it grew more quickly than the other late sub-adults. LAGs, that were sometimes double LAGs, were observed in the other early sub-adults (Table 2).

A prominent patch of Sharpey’s fibers was observed in juvenile MVD-R-11c (Fig. 4A). They were also seen in the early sub-adults and in the late sub-adults MVD-R-25 and MVD-R-26 in the mid- to outer cortex (Table 2).

### Tibiae

**Figure 5:**
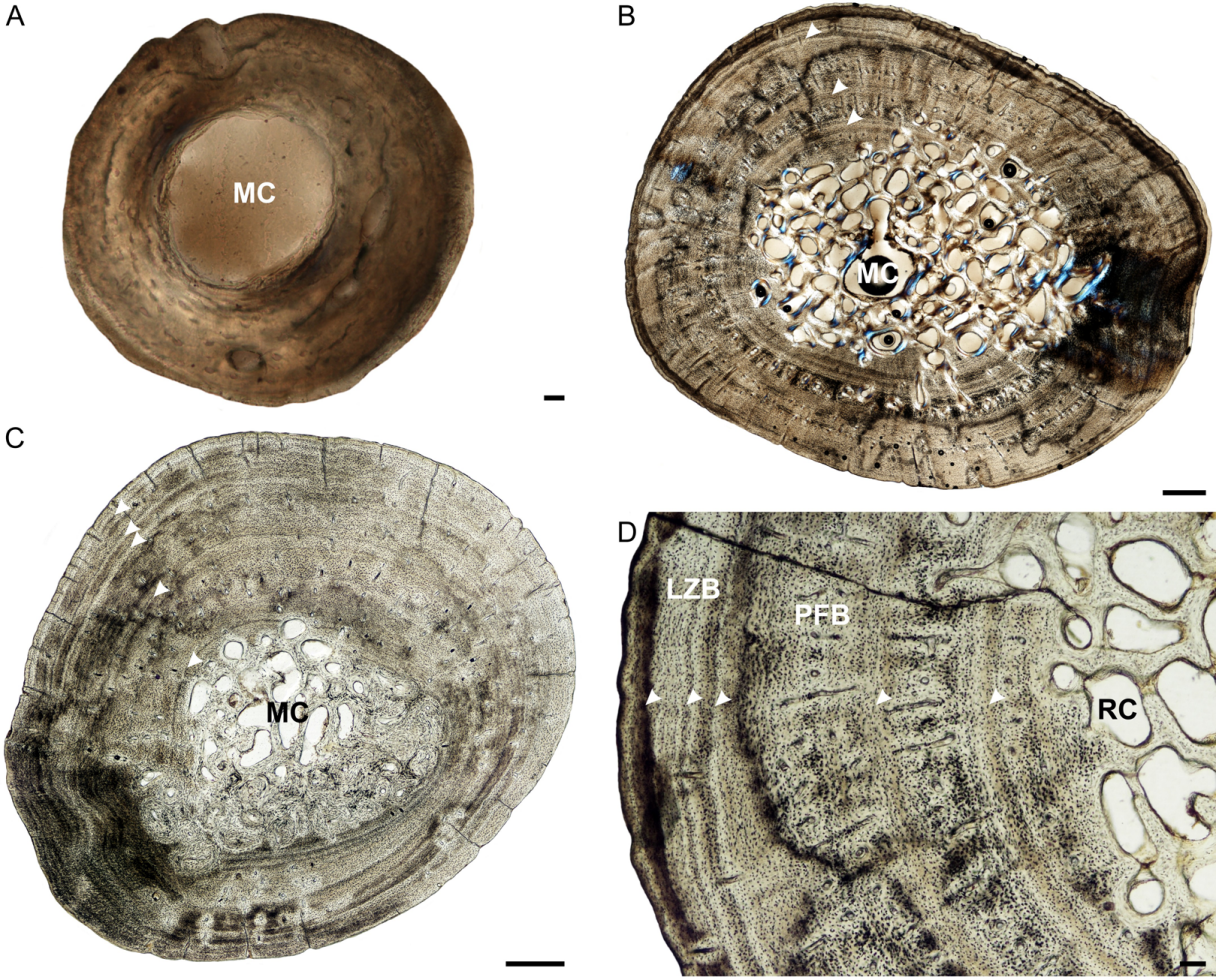
Tibia osteohistology of *Stigmochelys pardalis*. (A) Whole cross-section of juvenile MVD-R-12h in cross-polarized light showing a thick cortex. (B) Early sub-adult MVD-R-31a showing an almost infilled medullary cavity and a cortex of parallel-fibered bone interrupted by growth marks (arrowheads). (C) Late sub-adult MVD-R-33 showing a completely infilled medullary cavity. Arrowheads indicate annuli and LAGs. (D) High magnification of late sub-adult MVD-R-33 showing an inner region of parallel-fibered bone and outer region of lamellar-zonal bone. Arrowheads indicate LAGs. Abbreviations: LZB, lamellar-zonal bone; MC, medullary cavity; PFB, parallel-fibered bone; RC, resorption cavity. Scale bars equal 100 μm in (A) and (D), and 500 μm in (B) and (C).

The medullary cavities of the juvenile tibiae differ from one another. The medullary cavity of MVD-R-12 is open, almost centrally placed and circular, whereas MVD-R-11 has a larger medullary cavity that is partially infilled with bony trabeculae resulting in several smaller cavities. However, this, once again, could be a result of the small size of the bones and the major differences along the small diaphysis. The cortex of the juveniles is relatively thin (average *K* = 0.66). MVD-R-11 has a thin medullary cavity that varies in thickness in different regions of the bone resulting in an average Cg of 0.645. However, these differences may be due to the small size of the bones resulting in differences along the short diaphysis. This is seen in MVD-R-12, where a thick cortex is observed in MVD-R12h (Fig. 5A) but overall, a low average Cg of 0.635. There is only one early sub-adult, MVD-R-33 (Fig. 5B). The growth center is similar to that seen in the early sub-adult ulnae in that it is almost completely infilled with bony trabeculae and only a few resorption cavities are present in the peri-medullary region. This specimen has an extremely thick cortex with a *K* value of 0.14 and a high Cg of 0.987. The medullary cavities of the late sub-adults are small, centrally placed, generally oval and infilled with bony trabeculae (Fig. 5C). Cavities are relatively large throughout the medullary cavity but there are a number of smaller cavities towards the inner cortex creating a slightly gradual transition zone. Medullary cavities of older specimens are larger compared to younger specimens. The cortices of late sub-adults MVD-R-30, MVD-R-31a and MVD-R-32 are thick and remain so throughout the section (average *K* = 0.28). In contrast, the ontogenetically older MVD-R-2e and MVD-R-29 have thinner cortices, which vary in thickness around the bone (average *K* value of 0.62). Despite this, the Cg is approximately constant amongst the various specimens with an average of 0.91. Secondary osteons were first observed in the early sub-adult where they are sparse and limited to the medullary region. The late sub-adults exhibit more secondary osteons and resorption cavities in the perimedullary region and inner cortex. They are also more abundant in the younger late sub-adults compared to older individuals.

The bone tissues of the juveniles are similar to that seen in the other elements. Vascular canals are not particularly abundant, with a low average vascularization of 1.5% and are in the form of simple longitudinally-oriented canals. The bone tissue of the early sub-adult is lamellar-zonal with dense, organized and flattened osteocyte lacunae, interrupted by LAGs. The vascular canals are mostly simple, with a few anastomoses, and a few very small primary osteons (vascularization 0.06%). However, there are also thin radiating canals within the thickest part of the cortex. Vascularization decreases slightly at the periphery. The bones of the late sub-adults are comprised of lamellar-zonal-bone tissue with highly organized flattened or oval osteocyte lacunae in parallel rows throughout the cortex. There are slight deviations in MVD-R-30 and MVD-R-31a (Fig. 5D), where parallel-fibered bone tissue is present in the inner cortex of the bone. Vascular canals are small and mainly unbranched, but there are a number of branched simple canals with short anastomoses. Primary osteons are small, sparse, longitudinally-oriented and randomly distributed throughout the cortex. However, there are several circular rows in some of these individuals. As a result of the small size and limited number of canals and primary osteons, vascularization is poor in all specimens with an average of 0.1%.

Three LAGs were observed in the cortex of the early sub-adult, but the growth marks include both annuli and LAGs in the late sub-adults. In late sub-adult MVD-R-31a a triple annulus, two single annuli and two double LAGs were observed, whereas MVD-R-29 exhibits six LAGs and MVD-R-32 five annuli. Sharpey’s fibers were observed in late sub-adult specimens MVD-R-30, MVD-R-31a and MVD-R-32 (Table 2).

### Fibulae

**Figure 6:**
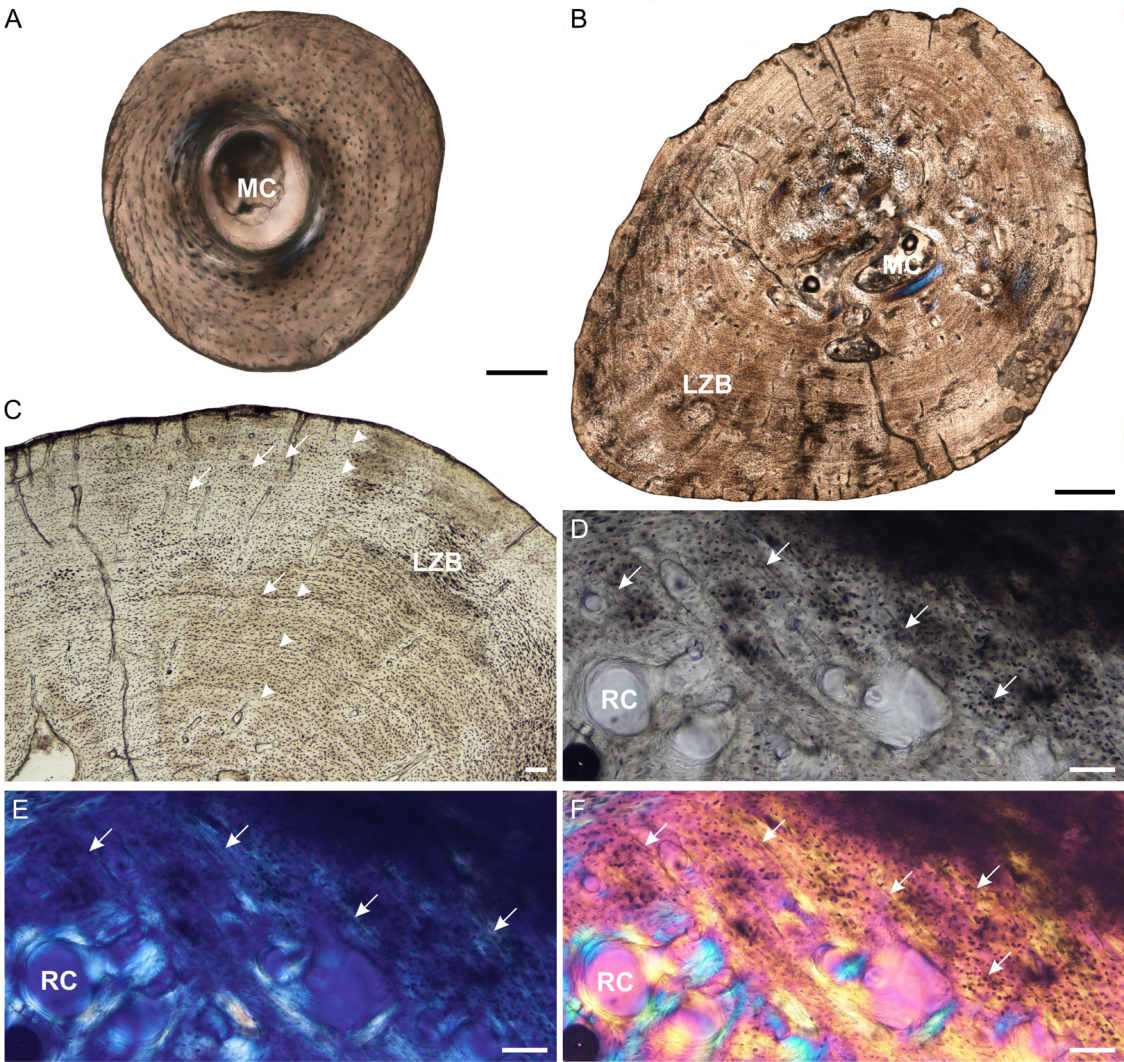
Fibula osteohistology of *Stigmochelys pardalis*. (A) Whole cross-section of juvenile MVD-R-11g showing a thick cortex. (B) Early sub-adult MVD-R-36 showing an infilled medullary cavity and a thick cortex of lamellar-zonal bone. (C) Late sub-adult MVD-R-36 showing faint annuli (arrowheads) and Sharpey’s fibers (arrows). (D–F) Late sub-adult MVD-R-2f showing Sharpey’s fibers (arrows). Arrowheads indicate LAGs. Abbreviations: LZB, lamellar-zonal bone; MC, medullary cavity; RC, resorption cavity. Scale bars equal 100 μm in (A), (C) and (D), and 500 μm in (B).

The medullary cavities of the juveniles are circular or oval and open (Fig. 6A), except for MVD-R-12k, which has small bony trabeculae separating the medullary cavity into five cavities. Overall, the juveniles have thin cortices (average = 0.67), possibly due to the section including part of the metaphysis (due to the tiny size of the bones, it was difficult to obtain the perfect cross-section through the mid-diaphysis). Juvenile specimen MVD-R-11f has a thick cortex (*K* = 0.33) and provides a good representation of the bone microanatomy of the juvenile fibula and will be the focus of the description here (Fig. 6A). This specimen also has a high Cg of 0.89 whereas MVD-R-12k and MVD-R-12j have an average Cg of 0.62. Early sub-adults are limited to a single specimen, MVD-R-36 which has an infilled circular medullary cavity (Fig. 6B). The cavity is surrounded by a number of smaller cavities, but the transition between compact and cancellous bone is still sharp. The early sub-adult cortex is thick throughout the section with a *K* value of 0.15 and a Cg of 0.957. The medullary cavities of the late sub-adults are all centrally placed and exhibit a variety of different shapes from diamond, rectangular to oval. Medullary cavities are all infilled with bony trabeculae. Larger cavities are concentrated towards the center and cavities decrease in size towards the periphery, creating a gradual transition zone. The cortices are thick (average *K* = 0.28) in the younger late sub-adults, but thinner in the ontogenetically oldest individual MVD-R-2f (*K* value = 0.55). As a result, MVD-R-2f has the lowest Cg of 0.822. Secondary osteons are first observed in the late sub-adults, but they are rare. However, resorption cavities are more numerous and concentrated in the peri-medullary region and inner cortex.

The juvenile bone tissues contain flattened to globular osteocyte lacunae aligned roughly in parallel to one another or randomly distributed around the cortex indicating a mixture of parallel-fibered and lamellar bone matrix. Vascularization is either absent or very low (MVD-R-12k = 0.87%) suggesting a slow growth rate. The bone tissue in the early sub-adult is lamellar-zonal-bone (Fig. 6C) with dense flattened osteocyte lacunae that are neatly organized in parallel rows and in certain areas, randomly distributed. Simple vascular canals are both branched and unbranched and randomly distributed throughout the cortex with a number of thin radiating canals in the thickest part of the cortex. There are a few small primary osteons, but they are rare and limited to the inner cortex. Vascularization is very low (0.07%) and further decreases towards the periphery indicating an overall decrease in bone growth. All late sub-adult specimens have a lamellar-zonal-bone tissue. Vascular canals are predominantly simple, small and unbranched and show a substantial decrease towards the periphery. As a result, vascularization is low (average 0.1%). A few longitudinally-orientated primary osteons are present, and in MVD-R-35, are arranged in circular rows. Growth marks are absent in the juveniles, but LAGs were observed in all individuals from the early sub-adult stage (Table 2).

MVD-R-11f shows a small region in cross polarized light that appears to be Sharpey’s fibers and may represent a site of muscle attachment. This differs from all other juvenile bones (except juvenile femur MVD-R-11c), where Sharpey’s fibers were not observed (Table 2). Sharpey’s fibers are present in all late sub-adult specimens (See Figs. 6D–6F for close ups of MVD-R 2f).

### Lifestyle

The lifestyle reading of the juvenile specimens mostly suggests an aquatic lifestyle, but juvenile humerus MVD-R-12a predicted a terrestrial lifestyle. Additionally, sections from juvenile MVD-R-11d and MVD-R-11e were taken closer to the epiphyses (due to the difficulty of sectioning such small bones), resulting in an incorrect lifestyle reading. Lifestyle readings consistently inferred an incorrect aquatic lifestyle for all the early sub-adult and late sub-adult specimens. However, one section from adult humerus MNHN-ZA-AC 2010-2 predicted an amphibious lifestyle, whereas another section predicted a terrestrial lifestyle. The first section was taken from nearer the proximal epiphysis (growth center) and the second from the midpoint of the diaphysis showing that the sampled region of the bone can affect the lifestyle reading (Table 3).

**Table 3:** Quantified microanatomical information gathered from Bone Profiler and used in the determination of inferred lifestyle.

Element	Accession #	Age class	*K*	Cg	Min	Max	*S*	*P*	Lifestyle reading
Humerus	MVD-R-12b	J	0.51	0.69	1.52	0.97	0.07	0.51	0
	MVD-R-12a	J	0.65	0.66	0.22	1.00	0.07	0.65	2
	MVD-R-11b	J	0.56	0.85	0.55	1.00	0.05	0.56	0
	MVD-R-11a	J	0.69	0.73	0.44	1.00	0.04	0.69	0
	MVD-R-10a	ESA	0.23	0.96	0.49	1.00	0.06	0.11	0
	MVD-R-9	ESA	0.44	0.97	0.87	1.00	0.04	0.44	0
	MVD-R-8	ESA	0.11	0.96	0.53	1.00	0.08	0.25	0
	MVD-R-7	ESA	0.40	0.96	0.76	1.00	0.04	0.40	0
	MVD-R-6	ESA	0.12	0.93	0.72	1.00	0.04	0.48	0
	MVD-R-5	LSA	0.44	0.90	0.57	1.00	0.05	0.44	0
	MVD-R-4	LSA	0.41	0.92	0.54	1.00	0.06	0.41	0
	MVD-R-3	LSA	0.57	0.82	0.49	1.00	0.08	0.57	0
	MVD-R-2a	LSA	0.64	0.85	0.63	1.00	0.04	0.64	0
	MVD-R-1	LSA	0.43	0.91	0.54	1.00	0.08	0.43	0
	MNHN-ZA-AC 2010-2 (xs 5)	A	0.39	0.88	0.50	0.98	0.11	0.39	1
	MNHN-ZA-AC 2010-2 (xs 13)	A	0.23	0.86	0.00	1.00	0.18	0.23	2
Radius	MVD-R-12c	J	0.75	0.42	0.02	0.75	1.13	1.00	0
	MVD-R-18a	J	0.59	0.81	0.04	0.59	0.47	1.00	0
	MVD-R-17	ESA	0.04	0.99	0.06	0.04	3.04	1.00	0
	MVD-R-16	LSA	0.32	0.94	0.05	0.32	0.46	1.00	0
	MVD-R-15	LSA	0.12	0.97	0.08	0.12	2.29	1.00	0
	MVD-R-14	LSA	0.47	0.91	0.16	0.16	1.28	1.00	0
	MVD-R-13	LSA	0.50	0.89	0.08	0.50	0.61	1.00	0
	MVD-R-2b	LSA	0.45	0.89	0.07	0.45	0.53	1.00	0
Ulna	MVD-R-12e	J	0.67	0.52	0.02	0.67	4.53	0.97	–
	MVD-R-12d	J	0.56	0.68	0.05	0.56	2.94	1.00	–
	MVD-R-18b	J	0.48	0.83	0.03	0.48	0.30	1.00	–
	MVD-R-10b	ESA	0.22	0.95	0.03	0.22	1.65	1.00	–
	MVD-R-23	ESA	0.10	0.92	0.17	0.10	6.46	1.00	–
	MVD-R-22	ESA	0.50	0.95	0.04	0.49	0.82	1.00	–
	MVD-R-21	LSA	0.52	0.89	0.08	0.52	0.62	1.00	–
	MVD-R-20	LSA	0.71	0.96	0.20	0.22	0.74	1.00	–
	MVD-R-19	LSA	0.77	0.84	0.06	0.77	0.74	1.00	–
	MVD-R-2c	LSA	0.70	0.80	0.04	0.70	0.61	1.00	–
Femur	MVD-R-12g	J	0.42	0.81	0.02	0.42	2.22	0.98	–
	MVD-R-12f	J	0.60	0.61	0.01	0.61	1.50	0.96	–
	MVD-R-11c	J	0.42	0.82	0.02	0.42	2.57	1.00	–
	MVD-R-10c	ESA	0.12	0.97	0.06	0.12	1.28	1.00	–
	MVD-R-28	ESA	0.38	0.96	0.04	0.38	0.73	1.00	–
	MVD-R-27	LSA	0.71	0.79	0.07	0.71	0.57	1.00	–
	MVD-R-26	LSA	0.59	0.91	0.04	0.59	0.75	1.00	–
	MVD-R-25	LSA	0.71	0.78	0.05	0.71	0.58	1.00	–
	MVD-R-24	LSA	0.64	0.80	0.06	0.64	0.53	1.00	–
	MVD-R-2d	LSA	0.67	0.75	0.05	0.67	0.46	1.00	–
Tibia	MVD-R-12i	J	0.71	0.49	0.02	0.71	7.27	1.00	0
	MVD-R-12h	J	0.44	0.78	0.02	0.44	1.70	0.97	0
	MVD-R-11d	J	0.73	0.56	0.04	0.73	0.18	1.00	2
	MVD-R-11e	J	0.75	0.73	0.02	0.75	0.53	1.00	2
	MVD-R-33	ESA	0.14	0.99	0.04	0.14	0.48	1.00	0
	MVD-R-32	LSA	0.40	0.89	0.04	0.40	0.36	1.00	0
	MVD-R-31a	LSA	0.19	0.92	0.13	0.19	1.16	1.00	0
	MVD-R-30	LSA	0.25	0.95	0.09	0.25	0.51	1.00	0
	MVD-R-2e	LSA	0.64	0.88	0.06	0.64	0.72	1.00	0
	MVD-R-29	LSA	0.60	0.91	0.06	0.60	0.75	1.00	0
Fibula	MVD-R-12k	J	0.65	0.72	0.05	0.65	0.36	1.00	–
	MVD-R-12j	J	0.69	0.52	0.01	0.69	0.35	1.00	–
	MVD-R-11f	J	0.33	0.89	0.02	0.33	2.57	1.00	–
	MVD-R-36	ESA	0.14	0.96	0.08	0.19	0.23	1.00	–
	MVD-R-31b	LSA	0.22	0.92	0.11	0.22	1.16	1.00	–
	MVD-R-35	LSA	0.46	0.93	0.05	0.46	0.67	1.00	–
	MVD-R-34	LSA	0.15	0.94	0.05	0.42	0.66	1.00	–
	MVD-R-2f	LSA	0.55	0.82	0.09	0.55	0.46	1.00	–

**Note:**

Abbreviations: MVD-R, Modern Vertebrate Database Reptiles; Xs, cross section; J, Juvenile; ESA, Early sub-adult; LSA, Late sub-adult; A, Adult K, the ratio of the internal diameter to the outer diameter of the bone; Cg, global compactness; Min, generated value for the minimum compactness; Max, value for the maximum compactness; S, the value proportional to the width of the transition zone between the medullary cavity and cortex; *P* the value of the transition area between the medullary cavity and compact cortex. 0-Aquatic; 1-Amphibious; 2-Terrestrial.

## Discussion

### Bone growth through ontogeny

*Stigmochelys pardalis* exhibits some relatively rapid growth during the juvenile stage as seen by the patches of woven-fibered bone. However, the presence of parallel-fibered bone in these specimens indicates relatively slower growth as well. This is highlighted by the fibulae, which contain a mixture of parallel-fibered and lamellar bone tissue. Parallel-fibered bone becomes the dominant bone tissue type through early to mid-ontogeny in all elements. However, radiating canals, indicating more rapid growth (De Margerie et al., 2004), were observed in the thickest areas of the cortex in the ulnae, tibiae and fibulae. These canals were found in both early and late sub-adults, but extended further towards the periphery in younger individuals and in certain cases appear to be associated with regions of muscle attachment to the bone. The presence of sub-reticular vascular canals deep in the cortex of the late sub-adult femur MVD-R-26 suggests that it grew more rapidly earlier in ontogeny (Stein, Hayashi & Sander, 2013). Primary osteons, indicating relatively rapid growth, are limited in most specimens and if present, are usually longitudinally oriented and randomly distributed throughout the cortex. However, these primary osteons were found in circular rows in the inner and mid cortices in a number of specimens, but particularly in the sub-adult humeri and ulnae. According to Rieppel (2000) the presence of vascular tissue is expected in testudines. Although generally absent in lepidosaurs, vascular bone tissues are present in varanid lizards (De Buffrénil, Houssaye & Bohme, 2008) and noted in marine iguanas (Hugi & Sánchez-Villagra, 2012). They are also generally present in archosaurs (including extant crocodilians) (Padian, Horner & De Ricqlès, 2004). The presence of primary osteons in *S. pardalis*, indicates similar growth rates to these taxa.

Growth marks were observed in all sub-adult and adult individuals. The deposition of growth marks indicates that growth temporarily decreased (annuli) or ceased (LAGs) during the unfavorable growing season and is typical of extant reptiles (Hutton, 1986).

The transition from parallel-fibered to relatively slower forming lamellar-zonal bone is typically found in older individuals and, together with a considerable decrease in vascularization towards the periphery and increase in number and predominance of LAGs, indicates a decrease in growth later in life (Chinsamy, 1990) and may indicate that sexual maturity had been reached (Hutton, 1986; Castanet, 1994; Cerda & Chinsamy, 2012; Köhler et al., 2012). The forelimb bone tissues of the early sub-adults display mostly annuli whereas late sub-adults display LAGs. This, together with the transition to lamellar-zonal bone, suggests that growth decreased notably later in development (Steyer et al., 2004).

An EFS consisting of multiple, closely spaced LAGs was observed in several late sub-adult humeri and fibulae, a radius, and the adult humerus. An EFS indicates a mature ontogenetic stage and often the onset of sexual maturity (Horner, De Ricqlès & Padian, 2000 in dinosaurs; De Ricqlès, Padian & Horner, 2001 in basal birds; Straehl et al., 2013 in xenarthran mammals and Klein, Scheyer & Tütken, 2009 in *Alligator mississippiensis*). Reproductive maturity in *S. pardalis* is typically reached within 10 years (Alexander & Marais, 2007). Humerus MVD-R-5 and fibula MVD-R-2f each contain six growth marks within the peripheral lamellar-zonal bone and radius MVD-R-14 contains four visible LAGs. The late sub-adults are between 56% and 60% of the adult size and thus, if the EFS noted in these individuals represents a true EFS, it shows extreme variability in this taxon. This variability has been noted in *S. pardalis* where conflicting results have been published (Lambert, 1995; Lambert, Campbell & Kabigumila, 1998; Mason et al., 2000). The age at which sexual maturity occurs is unclear. The variance within the species is likely a result of environmental fluctuations (Lambert, Campbell & Kabigumila, 1998) or captive vs wild specimens (Lapid, Nir & Robinzon, 2005; Ritz et al., 2010). Furthermore, the adult humerus in Nakajima, Hirayama & Endo (2014), MNHN-ZA-AC 2010-2, is of unknown origin. Therefore the large difference between the sizes of the late sub-adults is possibly a result of extreme differences in the growth rates between captive and wild specimens (as noted in Ritz et al., 2010). Thus, the presence of closely spaced growth marks at the sub-periosteal surface in smaller individuals is more likely to represent periods of harsh environmental conditions that are often seen in the Northern Cape and Free State regions (where the bones were collected), where extended periods of drought occur, and not the onset of sexual maturity.

Lamellar-zonal bone was more prevalent in the bones of the hind limb than the forelimb. These bones were considerably less vascularized than the forelimb, indicating relatively slower growth. Their vascular canals were small, mainly limited to the inner and mid cortices and decreased considerably in size and number towards the periphery, particularly in the tibiae and fibulae. As vascularization is closely linked to growth rate, the lower vascularization in the bones of the hind limb suggests that they grow more slowly than those of the forelimb. The humeri, radii and ulnae typically display annuli, indicating that growth did not cease completely during the unfavorable growing season, whereas the femur, tibia and fibula generally exhibit LAGs and even double LAGs, indicating that these bones stopped growing completely during the unfavorable growing season. This, together with a lower overall vascularization and predominance of lamellar-zonal bone in the hind limb elements, suggests that the hind limb grew more slowly than the forelimb, possibly due to the method of locomotion seen in terrestrial testudines.

Sharpey’s fibers are filamentous structures indicating areas where tendons or ligaments attach to bone (Chen et al., 2006). They are present in a number of specimens, as indicated in Table 3, but the extent and depth to which the fibers penetrate the bone tissues differs between elements and ontogenetic age. There is almost no evidence for attachment in the juveniles except in the femur MVD-R-11c where Sharpey’s fibers are present. Interestingly, this is the same bone that contains a growth mark. These features suggest that despite its similar size to the other juveniles it may have been slightly more developed. Sharpey’s fibers were observed less frequently in the humerus compared to the other elements, possibly because this element tends to undergo more extensive resorption, thus destroying any evidence of soft tissue attachment.

### Microanatomy and lifestyle

**Figure 7:**
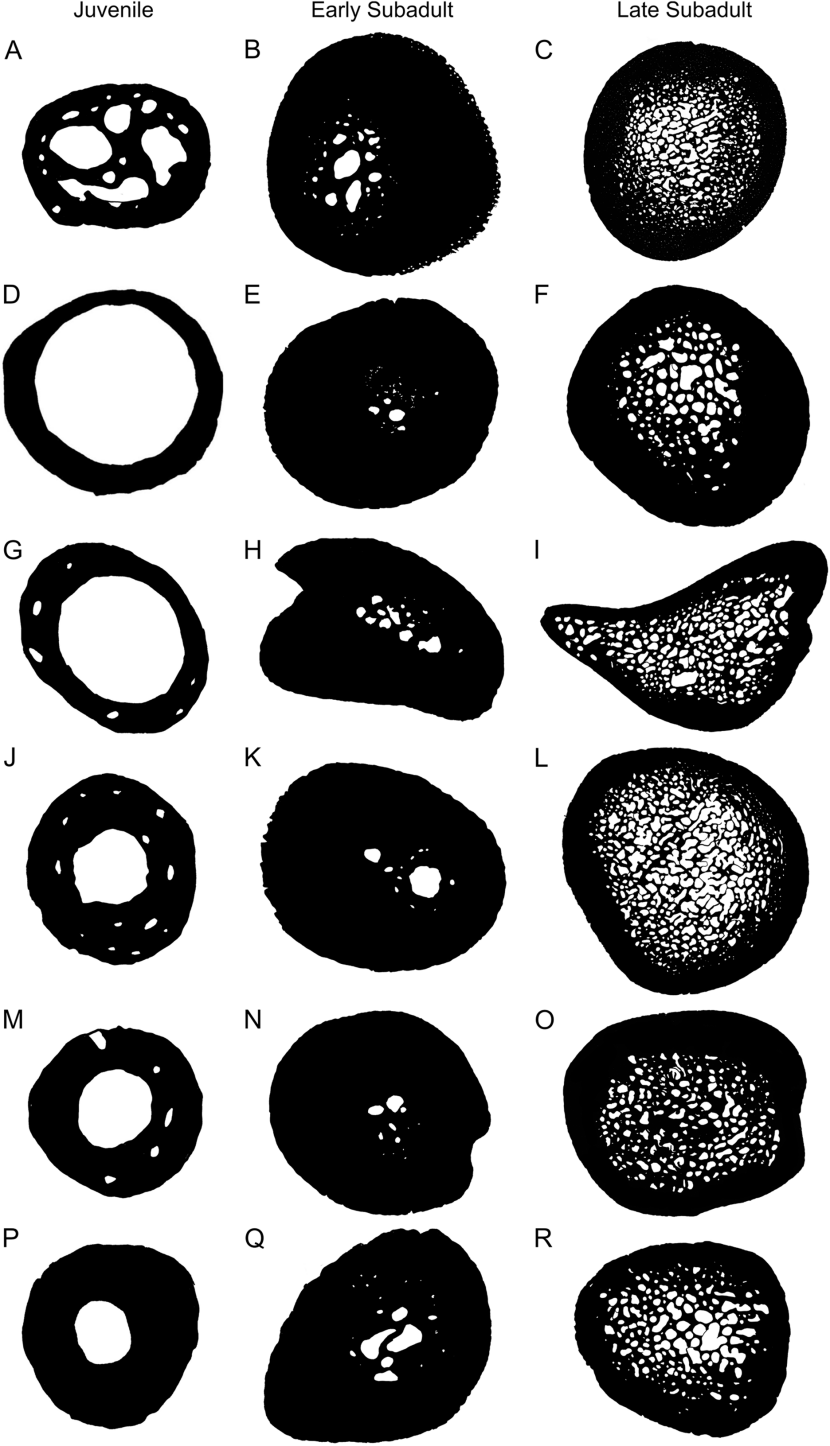
Representative microanatomy of juvenile, early sub-adult and late sub-adult *Stigmochelys pardalis* limb bones. (A) Juvenile humerus MVD-R-11a. (B) Early sub-adult Humerus MVD-R-10a. (C) Humerus MVD-R-2a. (D) Juvenile radius MVD-R-12c. (E) Early sub-adult radius MVD-R-17. (F) Late sub-adult radius MVD-R-2b. (G) Juvenile ulna MVD-R-12e. (H) Early sub-adult ulna MVD-R-22. (I) Late sub-adult ulna MVD-R-2c. (J) Juvenile femur MVD-R-12g. (K) Early sub-adult femur MVD-R-10c. (L) Late sub-adult femur MVD-R-2d. (M) Juvenile tibia MVD-R-12h. (N) Early sub-adult tibia MVD-R-33. (O) Late sub-adult tibia MVD-R-2e. (P) Juvenile fibula MVD-R-11f. (Q) Early sub-adult fibula MVD-R-36. (R) Late sub-adult Fibula MVD-R-2f. Not to scale.

One of the most noticeable changes through the various developmental stages is the variation in cortical thickness and size of the medullary cavity (Fig. 7). The cortices of juveniles are relatively thin and the bones contain open medullary cavities. During the early sub-adult stage the compact cortex thickens substantially while the medullary region becomes infilled with bony trabeculae and the actual main cavity occupies an even smaller portion of the bone. Relatively few resorption cavities surround the medullary cavity at this stage in most elements, but in the humerus there are a number of cavities particularly in the older early sub-adults. During the late sub-adult stage the thick cortex becomes extensively resorbed and the medullary cavity expands resulting in a large infilled medullary cavity surrounded by numerous resorption cavities and secondary osteons in the peri-medullary region. Larger cavities are concentrated towards the center of the medullary region and smaller cavities at the periphery resulting in a gradual transition zone between spongy and compact bone. We ran Linear Mixed Models to determine if our observations reflected a significant difference between early and late sub-adults, as indicated in the binarized images in Fig. 7. The test indicated a significant difference between early and late sub-adults for *K* (*p* = 3.59 × 10^−5^; df = 28.04) and a significant difference in the Cg between early and late sub-adults (*p* = 2.91 × 10^−5^; df = 31.27). Typically, larger animals display thicker cortices and smaller medullary regions (Canoville & Laurin, 2009), but this is not the case here. The extensive resorption seen in *S. pardalis* has been previously observed in other testudines (Scheyer & Sander, 2007; Nakajima, Hirayama & Endo, 2014). A large infilled medullary cavity has also been observed in testudine shell microstructure (Scheyer et al., 2014). Generally, a spongy infilled medullary cavity is found in aquatic species (such as cetaceans) where compact bone has been resorbed (De Buffrénil & Schoevaert, 1988), but this feature has been observed in terrestrial turtle species as well (Germain & Laurin, 2005; Kriloff et al., 2008; Nakajima, Hirayama & Endo, 2014). The medullary cavities of turtles are never free of bony trabeculae, irrespective of lifestyle as seen in Scheyer et al. (2014). Houssaye (2009) also noted that this is unusual for terrestrial species whereas Nakajima, Hirayama & Endo (2014) suggested it could be a result of their slow movement through an asymmetric gait used during locomotion. This asymmetric gait is seen in a number of species where the right and left limb display different patterns during movement. Alternatively, the trabecular infilling may be an attempt to provide extra support to the bones, which must support the extra weight of a shell during terrestrial locomotion.

Compactness has been used to infer the lifestyle of a variety of species through the use of Bone Profiler software to measure compactness (Germain & Laurin, 2005; Canoville & Laurin, 2010; Quemeneur, De Buffrénil & Laurin, 2013; Amson et al., 2014). However, the use of compactness profiles to exemplify lifestyle in testudines seems to be relatively inaccurate as noted by Scheyer et al. (2014). This observation is supported by the results in this study where all elements (apart from a few juveniles) indicate that *S. pardalis* is an aquatic species when in fact, it is purely terrestrial. Nakajima, Hirayama & Endo (2014) analyzed the humeral microanatomy of several aquatic and terrestrial turtle species and found that the growth center (and thickest part of the compact cortex) was often situated towards the proximal end of the bone and not at the mid-diaphysis level. They suggested that using a cross-section from the growth center would result in a different lifestyle reading to that shown in Canoville & Laurin (2010) and Germain & Laurin (2005). This is indeed the case as the humeral lifestyle inference model from Canoville & Laurin (2010) produced two separate lifestyle readings from the same specimen (adult specimen MNHN-ZA-AC 2010-2).

However, the humeri of aquatic species appear to grow more asymmetrically than terrestrial species as the growth center of many of the terrestrial species in Nakajima, Hirayama & Endo (2014) was generally in the same region of the thickest cortex located in the mid-diaphysis. A similar result was seen in the present study on *S. pardalis* where the thickest part of the compact cortex was found at the mid-diaphyseal level, the same region where Canoville & Laurin (2010) and Germain & Laurin (2005) would have analyzed the *S. pardalis* specimen in their study. Thus, at least for this species, the mid-diaphyseal level does represent the area of thickest compact cortex and thus, the best region to choose when inferring lifestyle in Bone Profiler.

## Conclusions

This is the first study to examine the inter-elemental and intra-elemental variation in the bone histology and microanatomy in a single species of testudine through ontogeny. The bone microstructure of *S. pardalis* is indicative of a slow growing species with either parallel-fibered bone or even slower growing lamellar-zonal bone being the predominant bone tissues from fairly early in ontogeny. An EFS, indicating a substantial decrease in growth rate, was observed in several late sub-adults and the adult humerus.

Notable inter-elemental histological variation is present between the forelimb and hind limb bones of *S. pardalis*. The bone tissues of the forelimb elements are predominantly parallel-fibered with slightly higher vascularization and interrupted by annuli, whereas the hind limb elements are less vascularized and reveal more lamellar-zonal bone interrupted by LAGs. These features indicate that the forelimb grew more quickly than the hind limb possibly as a result of the chelonian type of locomotion where the forelimb is slightly longer than the hind limb and thus, must grow more quickly to reach adult size.

Bone microanatomy revealed substantial primary bone deposition during early ontogeny with an onset of bone resorption and medullary cavity infilling during mid to late ontogeny. This caused the lifestyle inferences using traditional bone histological techniques to be inaccurate, as seen in previous studies. This shows that ontogeny and biomechanics do destroy the ecological signal in the bone microstructure of *S. pardalis* as there are major differences in the microanatomy between different age classes.

## Supplemental Information

10.7717/peerj.8030/supp-1 Supplemental Information 1 The storage site of all specimens used in the study.

## Additional Information and Declarations

### Data Availability

The following information was supplied regarding data availability: This is a descriptive study on bone histological thin sections. Highlighted histological and microanatomical features are illustrated in Figs. 1–7. Macro measurements of samples are available in Table 1. Results pertaining to growth marks and muscle attachment are present in Table 2. Quantified values used to generate lifestyle readings of each sample are available in Table 3. All specimens used in this study are housed at the National Museum, Bloemfontein, South Africa. The accession numbers for each specimen are available in the Supplemental File.

## References

[ref-1] Alexander G, Marais J (2007). A guide to the reptiles of southern Africa.

[ref-2] Amson E, De Muizon C, Laurin M, Argot C, De Buffrénil V (2014). Gradual adaptation of bone structure to aquatic lifestyle in extinct sloths from Peru. Proceedings of the Royal Society B: Biological Sciences.

[ref-3] Butcher MT, Blob RW (2008). Mechanics of limb bone loading during terrestrial locomotion in river cooter turtles (Pseudemys concinna). Journal of Experimental Biology.

[ref-4] Canoville A, Laurin M (2009). Microanatomical diversity of the humerus and lifestyle in lissamphibians. Acta Zoologica.

[ref-5] Canoville A, Laurin M (2010). Evolution of humeral microanatomy and lifestyle in amniotes, and some comments on palaeobiological inferences. Biological Journal of the Linnean Society.

[ref-6] Castanet J (1994). Age estimation and longevity in reptiles. Gerontology.

[ref-7] Cerda IA, Chinsamy A (2012). Biological implications of the bone microstructure of the Late Cretaceous Ornithopod dinosaur *Gasparinisaura cincosaltensis*. Journal of Vertebrate Paleontology.

[ref-8] Chen H, Yao XF, Emura S, Shoumura S (2006). Morphological changes of skeletal muscle, tendon and periosteum in the senescence–accelerated mouse (SAMP6): a murine model for senile osteoporosis. Tissue and Cell.

[ref-9] Chinsamy A (1990). Physiological implications of the bone histology of *Syntarsus rhodesiensis* (Saurischia: Theraposa). Palaeontologica Africana.

[ref-10] Crawford NG, Faircloth BC, McCormack JE, Brumfield RT, Winker K, Glenn TC (2012). More than 1000 ultraconversed elements provide evidence that turtles are the sister group of archosaurs. Biology Letters.

[ref-11] Crawford NG, Parham JF, Sellas AB, Faircloth BC, Glenn TC, Papenfuss TJ, Henderson JB, Hansen MH, Simison WB (2015). A phylogenomic analysis of turtles. Molecular Phylogenetics and Evolution.

[ref-12] Currey JD, Alexander RM (1985). The thickness of the walls of tubular bones. Journal of Zoology.

[ref-13] De Buffrénil V, Castanet J (2000). Age estimation by skeletochronology in the Nile monitor (*Varanus niloticus*), a highly exploited species. Journal of Herpetology.

[ref-14] De Buffrénil V, Houssaye A, Bohme W (2008). Bone vascular supply in monitor lizards (Squamata: Varanidae): influence of size, growth, and phylogeny. Journal of Morphology.

[ref-15] De Buffrénil V, Schoevaert D (1988). On how the periosteal bone of the delphinid humerus becomes cancellous: ontogeny of a histological specialisation. Journal of Morphology.

[ref-16] De Margerie E, Robin JP, Verrier D, Cubo J, Groscolas R, Castanet J (2004). Assessing a relationship between bone microstructure and growth rate: a fluorescent labelling study in the king penguin chick (*Aptenodytes patagonicus*). Journal of Experimental Biology.

[ref-61] De Ricqlès A, De Buffrénil V, Mazin JM, De Buffrénil V (2001). Bone histology, heterchronies and the return of tetrapods to life in water. Secondary Adaptation of Tertrapods to Life in Water.

[ref-17] De Ricqlès A, Padian K, Horner JR, Gauthier J, Gall LF (2001). The bone histology of basal birds in phylogenetic and ontogenetic perspectives. New Perspectives On the Origin and Early Evolution of Birds: Proceedings of the International Symposium in Honour of John H. Ostrum.

[ref-18] Drabik-Hamshare M, Downs CT (2017). Aspects of the home range ecology of the leopard tortoise in the semi-arid central karoo: an area threatened with fracking. Journal of Arid Environments.

[ref-19] Evers SW, Benson RBJ (2019). A new phylogenetic hypothesis of turtles with implications for the timing and number of evolutionary transitions to marine lifestyles in the group. Palaeontology.

[ref-20] Field DJ, Gauthier JA, King BL, Pisani D, Lyson TR, Peterson KJ (2014). Toward consilience in reptile phylogeny: miRNAs support an archosaur, not lepidosaur, affinity for turtles. Evolution & Development.

[ref-21] Francillon-Vieillot H, De Buffrénil V, Castanet J, Geraudie J, Meunier FJ, Sire JY, Zylberberg L, De Ricqlès A, Carter JG (1990). Microstructure and mineralisation of vertebrate skeletal tissues. Skeletal Biomineralisation: Patterns, Processes and Evolutionary Trends.

[ref-23] Germain D, Laurin M (2005). Microanatomy of the radius and lifestyle in amniotes (Vertebrata, Tetrapoda). Zoologica Scripta.

[ref-24] Girondot M, Laurin M (2003). Bone profiler: a tool to quantify, model, and statistically compare bone-section compactness profiles. Journal of Vertebrate Paleontology.

[ref-25] Heck CT, Varricchio DJ, Gaudin TJ, Woodward HN, Horner JR (2019). Ontogenetic changes in the long bone microstructure in the nine-banded armadillo (*Dasypus novemcinctus*). PLOS ONE.

[ref-26] Hill RV (2005). Integration of morphological data sets for phylogenetic analysis of amniota: the importance of integumentary characters and increased taxonomic sampling. Systematic Biology.

[ref-27] Horner J, De Ricqlès A, Padian K (2000). Long bone histology of the Hadrosaurid dinosaur *Maiasaura peeblesorum*: growth dynamics and physiology based on an ontogenetic series of skeletal elements. Journal of Vertebrate Paleontology.

[ref-28] Houssaye A (2009). “Pachystosis” in aquatic amniotes: a review. Integrative Zoology.

[ref-29] Hugi J, Sánchez-Villagra MR (2012). Life history and skeletal adaptations in the galapagos marine iguana (*Amblyrhynchus cristatus*) as reconstructed with bone histological data: a comparative study of Iguanines. Journal of Herpetology.

[ref-30] Hutton JM (1986). Age determination of living Nile crocodiles from the cortical stratification of bone. Copeia.

[ref-31] Klein N, Scheyer T, Tütken T (2009). Skeletochronology and isotopic analysis of a captive individual of *Alligator mississippiensis*, Daudin 1802. Fossil Record.

[ref-32] Köhler M, Marín-Moratalla N, Jordana X, Aanes R (2012). Seasonal bone growth and physiology in endotherms shed light on dinosaur physiology. Nature.

[ref-33] Kriloff A, Germain D, Canoville A, Vincent P, Sache M, Laurin M (2008). Evolution of bone microanatomy of the tetrapod tibia and its use in palaeobiological inference. Journal of Evolutionary Biology.

[ref-34] Lambert MRK (1995). On geographical size variation, growth and sexual dimorphism of the leopard tortoise, *Geochelene pardalis*, in Somaliland. Chelonian Conservation and Biology.

[ref-35] Lambert MRK, Campbell KLI, Kabigumila JD (1998). On the growth and morphometrics of leopard tortoise, *Geochelone pardalis*, in Serengeti National Park, Tanzania, with observations on effects of bushfires and latitudinal variation in populations of Eastern Africa. Chelonian Conservation and Biology.

[ref-36] Lapid RH, Nir I, Robinzon B (2005). Growth and body composition in captive *Testudo graeca terrestris* fed with a high-energy diet. Applied Herpetology.

[ref-37] Laurin M, Canoville A, Germain D (2011). Bone microanatomy and lifestyle: a descriptive approach. Comptes Rendus Palevol.

[ref-38] Lee MSY (1997). Pareiasaur phylogeny and the origin of turtles. Zoological Journal of the Linnean Society.

[ref-39] Lee MSY (2013). Turtle origins: insights from phylogenetic retrofitting and molecular scaffolds. Journal of Evolutionary Biology.

[ref-40] Legendre LJ, Botha-Brink J (2018). Digging the compromise: investigating the link between limb bone histology and fossoriality in the aardvark (*Orycteropus afer*). PeerJ.

[ref-41] Liu J, Rieppel O, Jiang D-Y, Aitchison JC, Motani R, Zhang Q-Y (2011). A new pachypleurosaur (Reptilia: Sauropterygia) from the lower middle triassic of southwestern China and the phylogenetic relationships of Chinese pachypleurosaurs. Journal of Vertebrate Paleontology.

[ref-42] Lyson TR, Rubidge BS, Scheyer TM, De Queiroz K, Schachner ER, Smith RMH, Botha-Brink J, Bever GS (2016). Fossorial origin of the turtle shell. Current Biology.

[ref-43] Mason MC, Kerley GIH, Weatherby CA, Branch WR (2000). Angulate and leopard tortoises in the thicket biome, Eastern Cape, South Africa: populations and biomass estimates. African Journal of Ecology.

[ref-44] Montoya-Sanhueza G, Chinsamy A (2017). Long bone histology of the subterranean rodent *Bathyergus suillus* (Bathyergidate): ontogenetic pattern of cortical bone thickening. Journal of Anatomy.

[ref-45] Nakajima Y, Endo H (2013). Comparative humeral microanatomy of terrestrial, semiaquatic and aquatic carnivorans using micro-focus CT scan. Mammal Study.

[ref-46] Nakajima Y, Hirayama R, Endo H (2014). Turtle humeral microanatomy and its relationship to lifestyle. Biological Journal of the Linnean Society.

[ref-48] Padian K, Horner JR, De Ricqlès A (2004). Growth in small dinosaurs and pterosaurs: the evolution of archosaurian growth strategies. Journal of Vertebrate Paleontology.

[ref-49] Quemeneur S, De Buffrénil V, Laurin M (2013). Microanatomy of the amniote femur and inference of lifestyle in limbed vertebrates. Biological Journal of the Linnean Society.

[ref-50] Reisz RR, Laurin M (1991). Owenetta and the origin of turtles. Nature.

[ref-51] Rieppel O (2000). Turtles as diapsid reptiles. Zoologica Scripta.

[ref-52] Rieppel O, Debraga M (1996). Turtles as diapsid reptiles. Nature.

[ref-63] Ritz J, Griebeler EM, Huber R, Clauss M (2010). Body size development of captive and free-ranging African spurred tortoises (Geochelone sulcata): high plasticity in reptilian growth rates. The Herpetological Journal.

[ref-60] Rose W (1950). The reptiles and amphibians of southern Africa.

[ref-53] Scheyer TM, Danilov IG, Sukhanov VB, Syromyatnikova EV (2014). The shell bone histology of fossil and extant marine turtles revisited. Biological Journal of the Linnean Society.

[ref-54] Scheyer TM, Sander PM (2007). Shell bone histology indicates terrestrial paleoecology of basal turtles. Proceedings of the Royal Society B: Biological Sciences.

[ref-55] Schmidt KP, Inger RF (1957). Living reptiles of the world.

[ref-56] Stein M, Hayashi S, Sander MP (2013). Long bone histology and growth patterns in Ankylosaurs: implications for life history and evolution. PLOS ONE.

[ref-57] Steyer JS, Laurin M, Castanet J, De Ricqlès A (2004). First histological and skeletochronological data on temnospondyl growth: palaeoecological and palaeoclimatological implications. Palaeogeography, Palaeoclimatology, Palaeoecology.

[ref-58] Straehl FR, Scheyer TM, Forasiepi AM, MacPhee RD, Sánchez-Villagra MR (2013). Evolutionary patterns of bone histology and bone compactness in Xenarthran mammal long bones. PLOS ONE.

[ref-62] Walker WF (1971). A structural and functional analysis of walking in the turtle, Chrysemys picta marginata. Journal of Morphology.

[ref-59] Wang Z, Pascual-Anaya J, Zadissa A, Li W, Niimura Y, Huang Z, Li C, White S, Xiong Z, Fang D, Wang B, Ming Y, Chen Y, Zheng Y, Kuraku S, Pignatelli M, Herrero J, Beal K, Nozawa M, Li Q, Wang J, Zhang H, Yu L, Shigenobu S, Wang J, Liu J, Flicek P, Searle S, Wang J, Kuratani S, Yin Y, Aken B, Zhang G, Irie N (2013). The draft genomes of soft-shell turtle and green sea turtle yield insights into the development and evolution of the turtle-specific body plan. Nature Genetics.

